# Integrated GC-MS and UPLC-ESI-QTOF-MS based untargeted metabolomics analysis of *in vitro* raised tissues of *Digitalis purpurea* L

**DOI:** 10.3389/fpls.2024.1433634

**Published:** 2024-08-22

**Authors:** Yashika Bansal, A. Mujib, Jyoti Mamgain, Rukaya Syeed, Mohammad Mohsin, Afeefa Nafees, Yaser Hassan Dewir, Nóra Mendler-Drienyovszki

**Affiliations:** ^1^ Cellular Differentiation and Molecular Genetics Section, Department of Botany, Jamia Hamdard, New Delhi, India; ^2^ Plant Production Department, College of Food and Agriculture Sciences, King Saud University, Riyadh, Saudi Arabia; ^3^ Research Institute of Nyíregyháza, Institutes for Agricultural Research and Educational Farm (IAREF), University of Debrecen, Nyíregyháza, Hungary

**Keywords:** metabolomics, indirect organogenesis, biochemical, SOD, POD, GC-MS, UPLC-ESI-QTOF-MS, cardenolides

## Abstract

*Digitalis purpurea* L. is one of the important plant species of Nilgiris, Kashmir and Darjeeling regions of India, belonging to the family Plantaginaceae, with well-known pharmacological applications. In the present investigation, an *in vitro* culture technique of indirect shoot organogenesis of *D. purpurea* is being explored; the biochemical attributes, the antioxidant activities and the metabolomic analyses were made by utilizing untargeted Gas Chromatography-Mass Spectrometry (GC-MS) and Ultra Performance Liquid Chromatography coupled with electronspray ionization/quadrupole-time-of-flight-mass spectrometry (UPLC-ESI-QTOF-MS) approaches. Initially, the leaf explants were used for callus induction and proliferation and maximum callusing frequency (94.44%) and fresh biomass (4.9 g) were obtained on MS, fortified with 8.8 µM BAP (6-benzyl amino purine) + 0.9 µM 2,4-D (2,4-dichlorophenoxyacetic acid), subsequently shoot formation (indirect organogenesis) was noted on the same MS medium with a shoot induction frequency of 83.33%. Later on, the biochemical and antioxidant potential of *in vivo*-, *in vitro* grown leaf and leaf derived callus were assessed. Significantly higher total phenol, flavonoid, DPPH (2,2-diphenyl-1-picrylhydrazyl), POD (peroxidase) and SOD (superoxide dismutase) activities were noticed in *in vitro* grown callus and leaf tissues compared with field grown leaf. The GC-MS analysis of each methanolic extract (*in vivo*-, *in vitro* derived leaf and leaf derived callus) displayed the presence of more than 75 bioactive compounds viz loliolide, stigmasterin, alpha-tocopherol, squalene, palmitic acid, linoleic acid, beta-amyrin, campesterol etc. possessing immense therapeutic importance. The UPLC-MS based metabolite fingerprinting of each methanolic extracts were conducted in both positive and negative ionization mode. The obtained results revealed variation in phytochemical composition in field - and laboratory grown tissues, indicating the impact of *in vitro* culture conditions on plant tissues. The detected phytocompounds belongs to various classes such as flavonoids, steroids, terpenoids, carbohydrates, tannins, lignans etc. The medicinally important metabolites identified were 20, 22-dihydrodigoxigenin, digoxigenin monodigitoxoside, apigenin, luteolin, kaempferide, rosmarinic acid, nepitrin and others. The results of the present study suggest that *in vitro* culture of *D. purpurea* could successfully be utilized for the novel drug discovery by producing such important phytocompounds of commercial interest in shorter duration without harming the plants’ natural population.

## 1 Introduction

For decades, medicinal plants have been a crucial source for pharmaceutical sector as more than half of the world’s populations are still reliant on traditional medicine system (Gomathi et al., 2013). Synthetically designed chemical drugs are often associated with multiple side effects, whereas plant-based medicines are derived naturally and thus, are more sustainable with negligible side effects (Konappa et al., 2020). Medicinal plants are frequently used in traditional medicine, food, cosmetics and healthcare as the plants are enriched with diverse groups of bioactive compounds which can be extracted for industrial and commercial purposes (Shakya, 2016; Rad et al., 2021). Plants produce a vast array of therapeutically important secondary metabolites like alkaloids, flavonoids, lignans, terpenoids, steroids and anthocyanins, along with significant primary metabolites like lipids, sugars and amino acids (Mickymaray, 2019; Thakur et al., 2019) play not only a decisive role in plant growth, development and reproductive cycle but also in defensive mechanisms (Singh et al., 2020). Several bioactive compounds exhibit crucial biological activities such as antineoplastic, anti-proliferative, anti-aging, anti-inflammatory, anti-angiogenic, anti-microbial, antiviral properties etc (Altemimi et al., 2017; Anand et al., 2019).

*Digitalis purpurea* L. (Plantaginaceae family) is an important biannual herbaceous plant with both ornamental and medicinal values and is commonly known as foxglove (Al-Oqab et al., 2022). The plant is indigenous to Europe and is grown in the Nilgiris, Kashmir and Darjeeling regions (Rehman Nengroo and Rauf, 2020). The well-known feature of *D. purpurea* is its tall spike-borne, campanulate flowers with color ranging from purple, pink, yellow, or white (Wu et al., 2012). It is currently a popular source of characteristic bioactive compounds cardenolides (digoxin, digitoxin, gitaloxin, gitoxin, strospeside); flavonoids (digicitrin and cyaniding); anthraquinone (digiferruginol), phenylethanoids (cornoside and maxoside) etc (Kreis, 2017; Amiri et al., 2023). These plant-based compounds exhibit multiple impact on health such as neuro-, hepato- and cardioprotective, anti-diabetic, anti-viral, anti-cancerous and cytotoxic activities (Verma et al., 2016a; Nartop et al., 2021). Digoxin and digitoxin are significant cardiotonic glycosides that are used to treat atrial arrhythmia and congestive heart failure (CHF) (Bhusare et al., 2018), by modifying the heart muscles’ contractile force and aid in their inotropic actions (Patel, 2016).

The evaluation of such phytocompounds as potent novel drugs requires simple efficient extraction, compound identification, economical production, employment of host organisms, conduction of preclinical, clinical trials and quantitative structure-activity relationship (QSAR) studies and complete metabolomic profiling of source plants (Dey et al., 2020). However, several factors restricting commercial production of these phytocompounds from plants growing in the wild include poor accessibility, over-utilization, cultivation challenges, low production, seasonal fluctuations, complications in extraction, extent of impurities, and the financial cost associated with suitable screening biological assays (Halder et al., 2019). In such scenario, micropropagation techniques prove to be beneficial in producing elite clones that synthesize adequate amount of phytocompounds in a shorter period of time without harming natural habitat (Ajithan et al., 2019; Erişen et al., 2020). *In vitro* regenerated plantlets can be produced by two fascinating biotechnological methods: somatic embryogenesis and organogenesis (Bansal et al., 2022). The *in vitro* production of bioactive compounds can be obtained from callus, cell suspension, shootlets, roots etc. and the whole process consists of two steps: (i) biomass aggregation and (ii) phytocompounds biosynthesis and extraction (Chandran et al., 2020).

After transcriptomics and proteomics, the study of metabolites-related events in living organisms, or metabolomics has emerged as the third significant area of functional genomics (Deepalakshmi et al., 2016). Thus, the integration of *in vitro* plant cultures with metabolomics investigations opens significant avenues to analyze the bioactive profile of different plant samples qualitatively and quantitatively (Carla Guimarães Sobrinho et al., 2022). Furthermore, metabolomic information will offer exceptional perspectives on underlying characteristics of plant phenotypes concerning development, physiology, tissue identity, resistance, biodiversity, and so forth (Hall et al., 2002). A wide range of analytical techniques including Gas Chromatography-Mass Spectrometry (GC–MS), Nuclear Magnetic Resonance (NMR) (Abdelsalam et al., 2017), Ultra Performance Liquid Chromatography-Mass Spectrometry (UPLC–MS) (Oliveira et al., 2018), Capillary Electrophoresis (CE) and High-Performance Liquid Chromatography-Mass Spectrometry (HPLC–MS) (Marchetti et al., 2019) have been employed for high-throughput analysis of metabolites over the past ten years.

Because of the extremely diverse biochemistry of plants, and presence of several important bioactive groups, both the GC-MS and LC-MS techniques are most frequently used in detecting critical phytocompounds (El Sayed et al., 2020). In metabolomics analysis, the MS is used in conjunction with GC or LC to leverage the advantages of each technique-the robustness of MS detectors and the higher resolution and reproducibility of chromatographic system. The GC-MS is often used for the study of volatile organic compounds, lipids and derivatizable compounds, whereas LC-MS is typically used for the investigation of mostly semi-polar metabolites (Zeki et al., 2020). These techniques have recently been applied to different *in vitro* raised plant tissues like *Leucojum aestivum, Saraca asoca* and *Pluchea lanceolata* (Spina et al., 2021; Mamgain et al., 2022; Vignesh et al., 2022).

Considering all these aspects, the information regarding the metabolic profiling of *in vitro* raised tissues of *D. purpurea* is still unknown, so it would provide insights about the plants’ phytochemistry and pharmacological importance. Thus, in the present work, we report the comparative and untargeted metabolite profiling of leaf-callus and *in vivo-* and *in vitro*-raised leaf tissues of *D. purpurea* by GC-MS and LC-MS techniques. The biochemical attributes such as total phenolic content, total flavonoid content and anti-oxidant activities were also assessed in cultured tissues during metabolite accumulation in *D. purpurea*. This is the first report of its kind which may auger novel new drug development in future.

## 2 Materials and methods

### 2.1 Chemicals and reagents

All the chemicals and solvents used in this study were of analytical grade. MS medium (Murashige and Skoog, 1962), methanol, Folin-ciocalteu reagent, Gallic acid, Quercetin, 2,2-Diphenyl-1-picrylhydrazy (DPPH), hydrogen peroxide, Triton X-100, ethylenediaminetetraacetic acid (EDTA), polyvinylpyrollidone (PVP), methionine, Nitro blue tetrazolium (NBT), riboflavin and all other chemicals and reagents were purchased from Himedia (Mumbai, India), SRL (Mumbai, India) and Sigma Aldrich (USA).

### 2.2 Explant preparation and *in vitro* culture conditions

Healthy and young leaves of *D. purpurea* were collected and underwent surface sterilization following the protocol of Bansal et al. (2022). Under sterilized conditions of laminar air flow, the leaf explants (3-4 cm) were inoculated on MS medium containing 3% (w/v) sucrose and 0.8% (w/v) agar. After adjusting the pH to 5.8, the medium was sterilized for 15 min at 121°C at 1.06 kg/cm^2^ of pressure. The culture was maintained at 24 ± 2°C in the dark for initial two days, and then switched to a 16:8h photoperiod cycle (cool white fluorescent light with an intensity of 50 μmol/m^2^/s^-1^).

### 2.3 Callus induction and indirect organogenesis

For callus induction, the leaf explants of *D. purpurea* were cultured onto MS medium supplemented with a combination of 6-benzylaminopurine (BAP) and 2,4-dichlorophenoxy-acetic acid (2,4-D) at varying concentrations. After 4 weeks, the produced callus was sub-cultured on the same medium for another 4 weeks to obtain shoots (indirect organogenesis). Each treatment included five replicates (one explant/test tube), and every experiment was conducted thrice. The callus induction percentage (%), fresh biomass (g) as well as indirect shoot induction percentage (%) and the mean shoot numbers/callus mass were recorded after 4 weeks period.

### 2.4 Biochemical analyses

#### 2.4.1 Sample preparation

The leaf derived calli and *in vivo* and *in vitro* (organogenic derived) grown leaf tissue of *D. purpurea* were collected and air dried at room temperature for 5 days. Using a mortar and pestle, around 1.0 g of each air-dried sample was crushed into a fine powder. Each sample was then separately extracted using 10 mL of methanol (MeOH) solvent on a rotary shaker for 2 days, followed by filtration of extracts with Whatman filter paper No. 1. The filtered samples were then centrifuged for five min at 12,000 rpm, and the recovered supernatant was stored at 4°C for further use.

#### 2.4.2 Total phenolic content estimation

The TPC determination was carried out with Folin-Ciocalteu protocol (Baliyan et al., 2022). Firstly, 2.5 mL of 10% (v/v) Folin-Ciocalteu (FC) reagent ((Sigma-Aldrich, New York, NY, USA) was thoroughly mixed with approximately 0.5 mL of extract and kept at room temperature (RT) for 5 min. After that, 2 mL of 7% Na_2_C0_3_ was added, followed by incubation for 90 min at RT. Then, the absorbance of each sample was measured at a wavelength of 765 nm by using a UV-Vis spectrophotometer (Biolinkk, BL-295, Delhi, India) against a blank. The experiment was conducted in triplicates and a calibration curve of gallic acid was prepared to determine the total phenolic content in each sample. Results were expressed as milligrams of Gallic acid equivalents per gram of Dry Weight (mg GAE/g DW).

#### 2.4.3 Total flavonoid content estimation

The TFC determination was conducted following the protocol reported by Aryal et al. (2019). The first step involved mixing 1.0 mL of extract (sample) solutions with 0.2 mL of 10% AlCl_3_ and 0.2 mL of 1 M potassium acetate solution. Later, 3.6 mL of distilled water was added to the total reaction volume, which was then allowed to incubate at room temperature for 30 min. After fully mixing the aforementioned solution, the absorbance at 510 nm was measured using a UV-visible spectrophotometer against a blank. Every TFC determination was carried out in triplicate and a calibration curve of quercetin was plotted to determine the total flavonoid content in each sample. Results were expressed as milligrams of Quercetin Equivalents per gram of Dry Weight (mg QE/g DW).

#### 2.4.4 DPPH radical scavenging activity assay

The free radical scavenging activity (FRSA) of extract samples of *D. purpurea* was analyzed using 2,2-diphenyl-1-picrylhydrazyl (DPPH) according to Baliyan et al. (2022) protocol. Approximately 100 µL of methanolic extracts (samples) were taken in separate test tubes and mixed with 3.0 mL DPPH (0.024% w/v). As a reference standard, 100 µL of methanol was mixed with 3.0 mL of DPPH. The samples were then incubated at room temperature for 90 min in complete darkness and the absorbance was checked at 517 nm wavelength. The following equation was used to determine the anti-oxidant potential of each sample (Khan H. et al., 2021):


$$\%\text{ Scavenging}=(A_{C}-A_{S}/A_{C})\times 100$$

where A_C_ = recorded absorbance of control and A_S_ = recorded absorbance of sample.

#### 2.4.5 Determination of peroxidase (POD; EC: 1.11.1.7) activity

The sample preparation and POD assay were carried out in accordance with Bansal et al.’s (2024) procedure. 10 mL of 0.1M phosphate buffer (pH=6.0) was taken to homogenize 1.0g of fresh samples (*in vivo*-, *in vitro*-raised leaf tissues and leaf derived callus). The extracts were then filtered, centrifuged at 12000 rpm for about 30 min at 4°C and the supernatants were collected. The samples were preheated at 65°C for 50 secs and then refrigerated until needed. A reaction mixture containing 1.0 mL of 10 mM K-phosphate buffer (pH = 7.0), 500µL of 1% guaiacol solution, 500µL hydrogen peroxide solution (0.4%), 500µL of enzyme extract, and 2.5 mL of distilled water was used for the peroxidase enzyme assay. The control was prepared with all the above stated reagents, except the enzyme extract. The development of tetraguaiacol was then confirmed by measuring the increase in absorbance at 470 nm within 30 min. The following formula was used to determine the enzymatic activity:


A=ELC

Wherein A = absorbance, E = extinction coefficient (6.39 mM^−1^cm^−1^), L = path length (1.0 cm) and C = enzyme concentration (mM/g FW), and FW = fresh weight of samples.

#### 2.4.6 Determination of superoxide dismutase (SOD; EC: 1.15.1.1) activity

Following the protocol provided by Mujib et al. (2022), the enzyme extract preparation and SOD assay were performed. Initially, 1.0 g of all the three fresh tissue samples were homogenized in 10 mL of 0.5 M sodium phosphate buffer (pH 7.3), containing 1.0% (v/v) Triton X-100, 3.0 mM ethylenediaminetetraacetic acid (EDTA), and 1.0% (w/v) polyvinylpyrollidone (PVP), to prepare the enzyme extracts. Finally, the supernatant was collected after the homogenate was filtered and centrifuged for 15 min at 4°C at 11,800 x g.

The SOD assay was conducted using a final reaction mixture of 3.0 mL, consisting of 50 mM K-phosphate buffer (pH 7.8), 45 µM methionine, 1.0 M Na_2_CO_3_, 2.25 mM Nitro blue tetrazolium (NBT) solution, 3.0 mM EDTA, 10 µM riboflavin, 10 µL of enzyme extract, and distilled water. A control group was established in which no enzyme extract was included. Subsequently, the mixture was subject to incubation at a temperature of 25°C for a duration of 10 min, while being exposed to fluorescent lamps with a power output of 15 W. The spectrophotometer was used to measure the absorbance of each sample at a wavelength of 560 nm. One unit of superoxide dismutase (SOD) activity is defined as the amount of enzyme required for 50% inhibition of NBT reduction. The activity is measured in units (U) per milligram of fresh weight (mg FW).

### 2.5 Metabolomics study using untargeted GC-MS approach

#### 2.5.1 Preparation of extracts

For GC-MS analyses, approximately 1 g of each air-dried leaf tissue (*in vivo*- and *in vitro* grown) and air-dried leaf derived callus was finely grounded with a mortar and pestle. Each powdered sample was then individually macerated with MeOH at room temperature for 48h on an orbital shaker. The resulting extracts were filtered using Whatman No. 1 filter paper and centrifuged at 10,000 rpm for 5 min. Later, the supernatants were filtered using a syringe filter (0.22 μm, Genetix, New Delhi, India) and stored at 4°C for metabolite analyses.

#### 2.5.2 GC-MS instrumentation and data analyses

The methanolic extracts of the samples were analyzed by GC-MS-QP-2010 equipment (Shimadzu, Tokyo, Japan), with the following parameters: The GC-MS separation was performed using the Rxi-5Sil MS GC Capillary Column (30 m, 0.25 mm ID, 0.25 μm film thickness). Helium was employed as the carrier gas at a consistent flow rate of 1.21 mL min−1. A GC-MS detection method utilized an electron impact ionization mode with ionization energy of 70 eV. The inlet temperature was set to 260°C, initial oven temperature was adjusted to 80°C and was programmed to increase to 280°C (hold time of 18 min) with a sample injection volume of 1 μl and scanning range of 40-600 m/z. For GC-MS analysis, the identification of phytocompounds present in each sample was done by comparing their retention times, peak area and peak area % to those of authenticated compounds listed in the database of NIST (National Institute of Standards and Technology) using GCMS solution software (Version 4.45 SP 1).

### 2.6 UPLC-ESI-QTOF-MS based metabolites profiling

#### 2.6.1 Sample preparation and metabolite extraction

The samples were prepared by shade drying each plant material (*in vivo*-, *in vitro* grown and leaf derived calli) of *D. purpurea* for 10-12 days. The shade dried materials were then finely grounded into powder and 50 mg per sample was utilized for metabolites extraction for LC-MS analysis. The powdered samples were extracted with 1.0 mL methanol (MeOH), sonicated at 40 kHz for 15 min at room temperature, filtered using a 0.22µm syringe filter (Sigma-Aldrich, USA) and centrifuged at 14000 rpm for 10 min at 4°C. The supernatants were collected in glass vials and subjected to UPLC-ESI-QTOF-MS analyses.

#### 2.6.2 LC-MS instrumentation

The determination of bioactive compounds in each sample (*in vivo*-, *in vitro*- derived callus and *in vivo* leaf tissues) was done by using UPLC-ESI-QTOF-MS technique. The analyses were performed by using a 2D nano ACQUITY Ultra-Performance Liquid Chromatography (UPLC) system (Waters Corporation, Milford, USA) online coupled with a SYNAPT G2-Si mass spectrometer (Waters Corporation, Milford, USA) via a NanoLockSpray dual electrospray ionization (ESI) source (Waters Corporation, Milford, USA). The separation of the bioactive compounds was carried out on acquity UPLC BEH C18 column (50x2.1mm, 1.7μm) operated at 35°C.The mobile phase comprised of two solvents: (A) 0.1% formic acid in water, and (B) acetonitrile. Gradient elution was done at a flow rate of 0.3 ml/min at room temperature and elution profile was represented in **Table 1**. The diode array detector was set at a wavelength range of 214-254 nm and the sample injection volume was 2 μl. The collision gas used was ultrahigh pure nitrogen gas. The mass data were acquired in both positive (+) and negative (-) electrospray ionization (ESI) modes with a scan range from m/z 100 to 1200 Da. The optimized parameters for positive mode were as follows: duration of the run was 25 min including 1766 cycles (0.4 secs each). For MS1 acquisition, ion spray voltage was set to 2500 V; turbo spray temperature of 500°C; collision gas, medium; nebulizer gas (GS1), heater gas (GS2) and curtain gas (CUR) rates were 50, 50 and 25 psi, respectively. For MS2 acquisition, a declustering potential of 80V; collision energy ranges from 20 to 45eV; and collision energy spread of 20V was applied as well. Negative ion mode had the same parameters but with an ion spray voltage of -2500 V.

**Table 1:** Gradient elution profile used in UPLC-ESI-QTOF-MS.

Time (min)	Mobile Phase-A (%)	Mobile Phase-B (%)
0.0	95.0	5.0
2.0	95.0	5.0
10.0	65.0	35.0
16.0	40.0	60.0
18.0	15.0	85.0
22.0	90.0	10.0
25.0	90.0	10.0

#### 2.6.3 Data processing and identification of secondary metabolites

In LC-MS approach, each obtained peak was examined in relation to the Human Metabolome Database (HMDB, http://www.hmdb.ca/) and METLIN (http://metlin.scripps.edu/) databases. In order to identify secondary metabolites, masses were matched within a 500ppm mass accuracy range in the respective libraries. In addition, certain metabolites were identified by comparing the reported MS/MS spectra with their m/z values in the total ion count (TIC) profile. In the positive ionization mode, three parent ion adducts of [M+H]^+^,[M+Na]^+^ and [M+H-H_2_O]^+^; and in negative ionization mode, only one parent ion adduct of [M-H]^-^ were considered in the databases search. The sturdiness of the compounds’ identification was validated using a comparison of the fragment masses obtained from the MS-MS spectra of every metabolite.

### 2.7 Statistical analysis

The *in vitro* culture experiment was conducted in a completely randomized manner in triplicates with six explants per treatment. The biochemical data were also statistically analyzed in triplicates. These data sets were presented as mean ± standard error. The statistical differences were tested using One-way ANOVA, followed by the *post hoc* analysis via Duncan’s Multiple Range Test (DMRT) (SPSS software, ver. 26.0) at p < 0.05 level.

## 3 Results

### 3.1 Callus induction and indirect organogenesis

**Figure 1:**
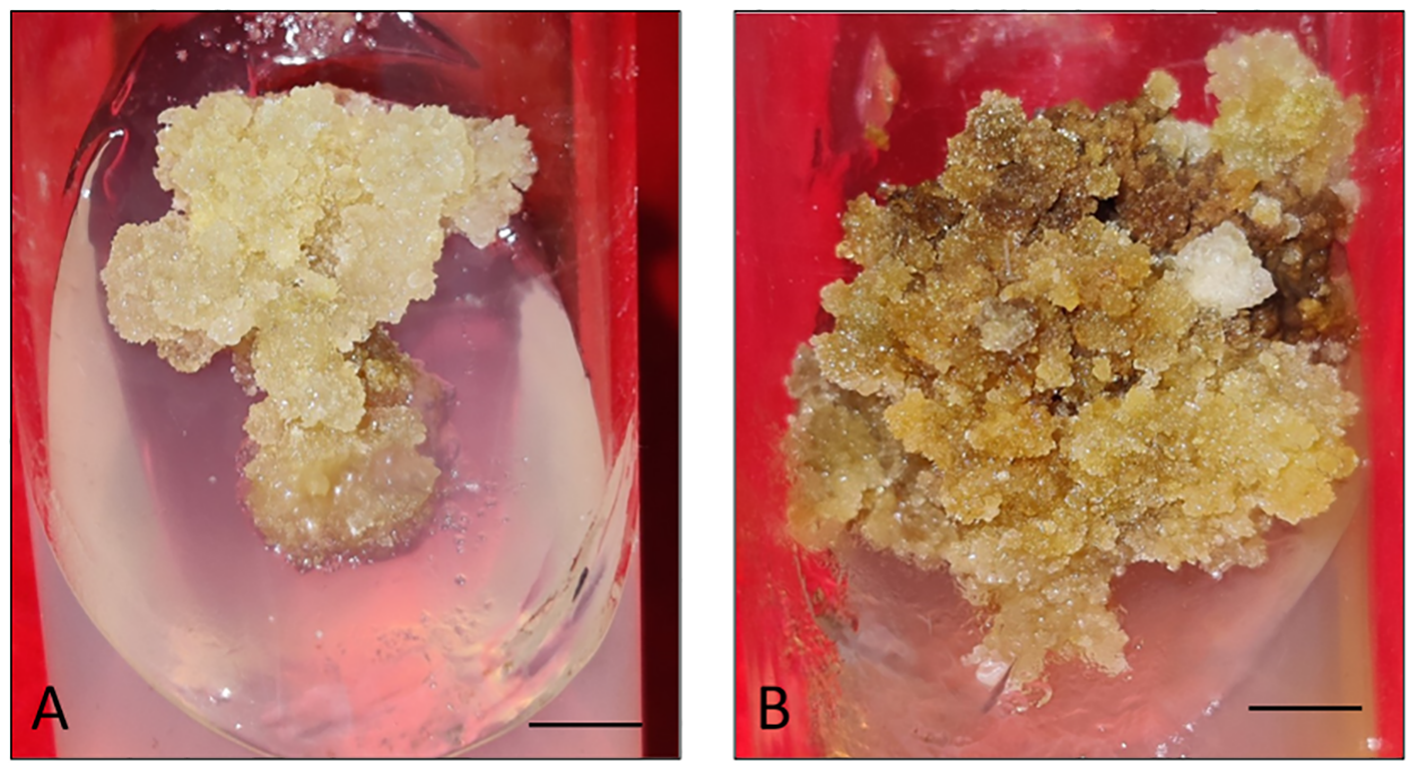
**(A, B)** Callus induction and proliferation from leaf explant of *D. purpurea* L. after 4 weeks of inoculation. (bars= 0.5 cm).

**Figure 2:**
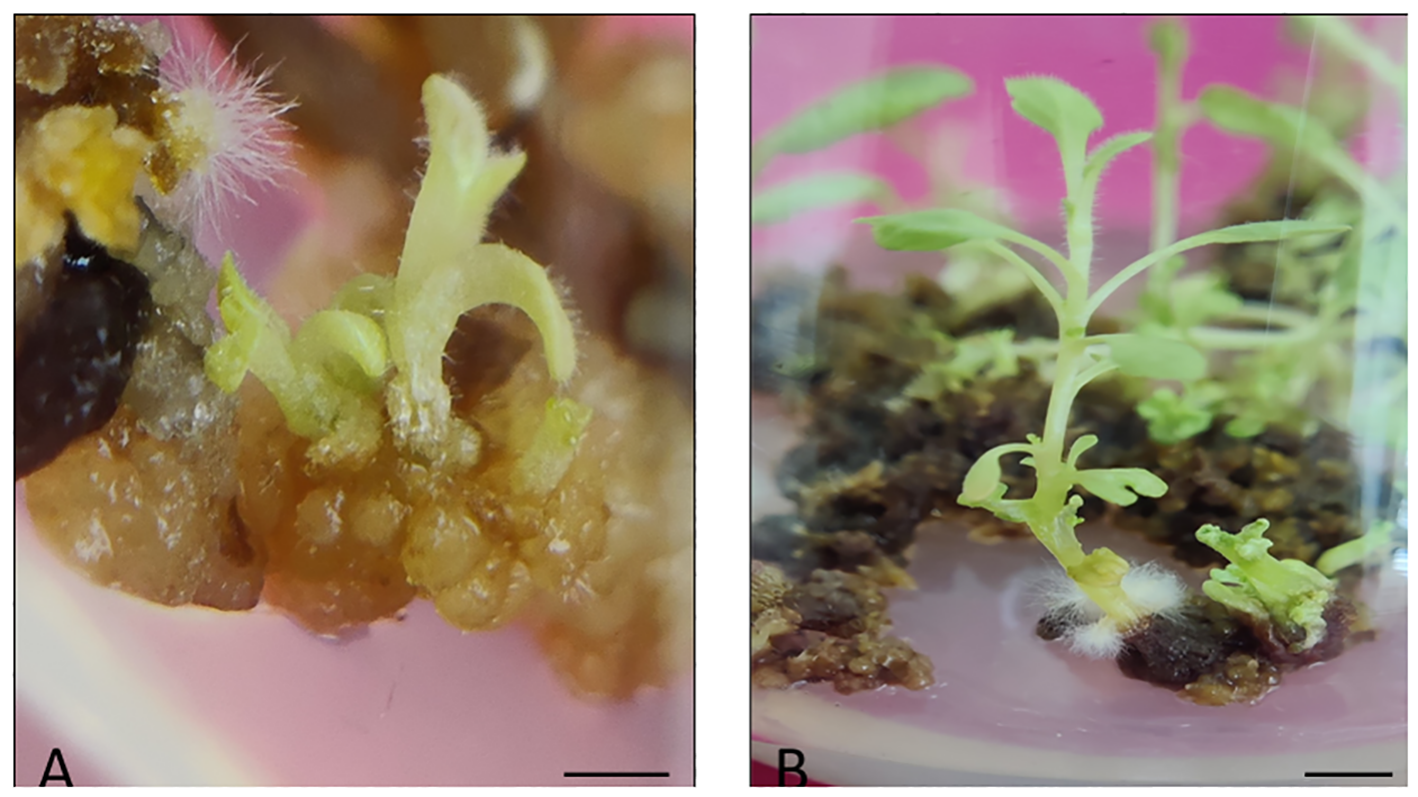
**(A)** Indirect shoot induction from leaf derived callus of *D. purpurea* L. after 4 weeks of sub-culturing (bar= 0.5 cm), **(B)** Elongation of indirect shootlet of *D. purpurea* L. on the same MS medium (bar= 1.0 cm).

Firstly, the healthy green leaf tissue of *D. purpurea* was employed as explants for callus induction. The induction was conducted on MS fortified with different concentrations of BAP and 2,4-D. Within about two weeks of culture, earliest signs of callus formation was noticed in all the tested media (**Table 2**). The calli were observed to be friable, with color varying from milky white to pale yellow (**Figures 1A, B**). All the tested media produced callus, with an overall rate of callus induction ranging from 94.44% to 22.21%. The best PGR combination was found to be 8.8 µM BAP and 0.9µM 2,4-D in which 94.44% callus induction and 4.9 g fresh biomass were noticed. This was followed by 8.8 µM BAP + 2.3µM 2,4-D and 4.4 µM BAP + 9.0 µM 2,4-D, which induced callus at the rates of 77.77 and 61.10 respectively. The lowest callusing frequency (22.21%) as well as fresh biomass (1.8 grams) was noted at 0.88µM BAP + 2.3µM 2,4-D. The sub-culturing of obtained callus on the same PGR amended MS medium resulted in shoots formation, i.e. indirect shoot organogenesis (**Figures 2A, B**). All the media produced plantlets on callus surface within 3-4 weeks of sub-culturing (**Table 3**). The highest shoot induction response was found to be 83.33% with maximum 4.7 ± 0.3 shoot numbers/callus mass was noted on BAP (8.8µM) and 2,4-D (0.9µM) added MS medium. On increasing 2,4-D concentrations, the rate of organogenesis gradually reduced. The lowest shoot development ability was observed at 0.88 µM BAP + 2.3µM 2,4-D, showing 11.10% shoot induction rate with 1.5 ± 0.3 mean shoot number/callus mass.

**Table 2:** Effect of different concentrations of BAP and 2,4-D on callus induction from leaf explants of *D. purpurea* L. after 4 weeks of inoculation.

PGRs Concentration (µM) BAP + 2,4-D	Callusing frequency (%)	Callus biomass (g)
(Control) 0	0^d^	0^d^
0.88 + 2.3	22.21 ± 5.56^c^	1.8 ± 0.7^c^
2.2 + 4.5	27.77 ± 5.56^c^	2.6 ± 0.6^bc^
4.4 + 9.0	61.10 ± 5.55^b^	3.6 ± 0.8^ab^
8.8 + 2.3	77.77 ± 11.11^ab^	4.1 ± 0.5^ab^
8.8 + 0.9	94.44 ± 9.62^a^	4.9 ± 0.4^a^

Each value is represented as mean ± standard error of three repeated experiments (n=6). Mean values with different superscripts within a column are significantly different from each other according to DMRT at p ≤ 0.05 level.

**Table 3:** Effect of BAP and 2,4-D on indirect organogenesis from leaf and hypocotyl explants of *D. purpurea* L. after 4 weeks of inoculation.

PGRs Concentration (µM) BAP+2,4-D	Indirect organogenesis rate (%)	Mean shoot no./callus mass
(Control) 0	0^d^	0^d^
0.88 + 2.3	11.10 ± 5.55^d^	1.5 ± 0.3^c^
2.2 + 4.5	33.33 ± 9.62^c^	2.1 ± 0.7^c^
4.4 + 9.0	38.89 ± 5.56^c^	2.5 ± 0.8^bc^
8.8 + 2.3	61.10 ± 5.55^b^	3.8 ± 0.1^ab^
8.8 + 0.9	83.33 ± 9.62^a^	4.7 ± 0.3^a^

Each value is represented as mean ± standard error of three repeated experiments (n=6). Mean values with different superscripts within a column are significantly different from each other according to DMRT at p ≤ 0.05 level.

### 3.2 Biochemical analyses

#### 3.2.1 Total phenolic content

The total phenolic content of MeOH extract of each sample was determined spectrophotometrically and the results are presented in **Table 4**. The TPC was found to be higher in *in vitro* developed callus and leaf samples than that of *in vivo* leaf sample. *In vitro* derived callus of *D. purpurea* had the highest phenolic content (5.43 ± 0.11 mg GAE/g DW), followed by *in vitro* regenerated leaf extract (4.47 ± 0.04 mg GAE/g DW) and *in vivo* derived leaf (3.64 ± 0.10 mg GAE/g DW). The results indicated that the callus sample accumulated more phenols than the other tested plant tissues.

**Table 4:** Total phenolic content (TPC) and total flavonoid content (TFC) of callus and leaf tissues of *D. purpurea* L.

Sample	TPC (mg GAE/g DW)	TFC (mg QE/g DW)
*In vivo* leaf	3.64 ± 0.10^c^	1.08 ± 0.16^c^
*In vitro* leaf	4.47 ± 0.04^b^	3.27 ± 0.01^a^
Leaf derived callus	5.43 ± 0.11^a^	2.52 ± 0.06^b^

TPC: total phenolic content, TFC, total flavonoid content; GAE, gallic acid equivalent; QE, quercetin equivalent; DW, dry weight. Each value is represented as mean ± standard error of three repeated experiments. Mean values with different superscripts within a column are significantly different from each other according to DMRT at p ≤ 0.05 level.

#### 3.2.2 Total flavonoid content

The total flavonoid contents in different tissue samples of *D. purpurea* were quantified (**Table 4**). The results were as follows: *in vitro* leaves (3.27 ± 0.01 mg QE/g DW) > leaf derived callus (2.52 ± 0.06 mg QE/g DW) > *in vivo* leaves (1.08 ± 0.16mg QE/g DW). The content of TFC in *D. purpurea* leaves regenerated *in vitro* was the highest, showing a two-fold increment than the *in vivo* grown leaf tissues. The flavonoid content of callus extract was moderate in level.

#### 3.2.3 DPPH radical scavenging activity assay

The methanolic extracts of *in vivo*-, *in vitro* raised leaf and leaf-callus were examined to determine the anti-oxidant potential using the DPPH assay. Results are summarized in **Table 5**. Both the *in vitro* raised tissues (callus and leaf) exhibited good anti-oxidant activity than the field grown leaf sample. The *in vitro* derived callus revealed the best free radical scavenging activity of 70.68 ± 0.57%, followed by *in vitro* regenerated leaf sample (60.59 ± 0.19%) and field grown leaf tissue (26.67 ± 1.11%). Thus, the lowest scavenging activity was displayed by *in vivo* grown leaf tissue.

**Table 5:** DPPH, POD and SOD activities of *in vivo*-, *in vitro*- leaf and callus samples of *D. purpurea* L.

Sample	DPPH Scavenging activity (%)	POD assay (mM/g FW)	SOD assay (U/mg FW)
*In vivo* leaf	26.67 ± 1.11^c^	0.53 ± 0.01^c^	0.53 ± 0.02^c^
*In vitro* leaf	60.59 ± 0.19^b^	0.60 ± 0.01^b^	1.13 ± 0.01^b^
Leaf derived callus	70.68 ± 0.57^a^	0.80 ± 0.03^a^	1.52 ± 0.01^a^

DPPH, 2,2-diphenyl-1-picrylhydrazyl; POD, peroxidase; SOD, superoxide dismutase; FW, fresh weight. Each value is represented as mean ± standard error of three repeated experiments. Mean values with different superscripts within a column are significantly different from each other according to DMRT at p ≤ 0.05 level.

#### 3.2.4 Activities of peroxidase and superoxide dismutase

The POD activity of *in vivo-, in vitro* grown leaf and leaf -callus sample of *D. purpurea* was assayed. It was observed that the POD activity was the highest in callus sample (0.80 ± 0.03 mM/g FW) than the other tested samples (**Table 5**). There is a minor difference in peroxidase activity in *in vitro* and *in vivo* derived leaf samples (0.60 ± 0.01mM/g FW and 0.53 ± 0.01 mM/g FW, respectively). The peroxidase activity thus showed this order: leaf derived callus > *in vitro* raised leaf > *in vivo* grown leaf. SOD assay was conducted to measure the anti-oxidant potential of each sample in *D. purpurea*. In this assay, a significant amount of anti-oxidant activity was noticed in all the extracts. The results revealed that the callus extract showed the maximum NBT degradation activity i.e. 1.52 ± 0.01 Units/mg FW (**Table 5**), while *in vitro* leaf displayed intermediate response of 1.13 ± 0.01 Units/mg FW, and the lowest activity (0.53 ± 0.02 Units/mg FW) was noted in *in vivo* grown leaf. Thus, the observation suggests that the highest SOD activity was observed in callus extract, followed by *in vitro* - and *in vivo* grown leaf tissue.

### 3.3 Metabolomics study using untargeted GC-MS approach

**Figure 3:**
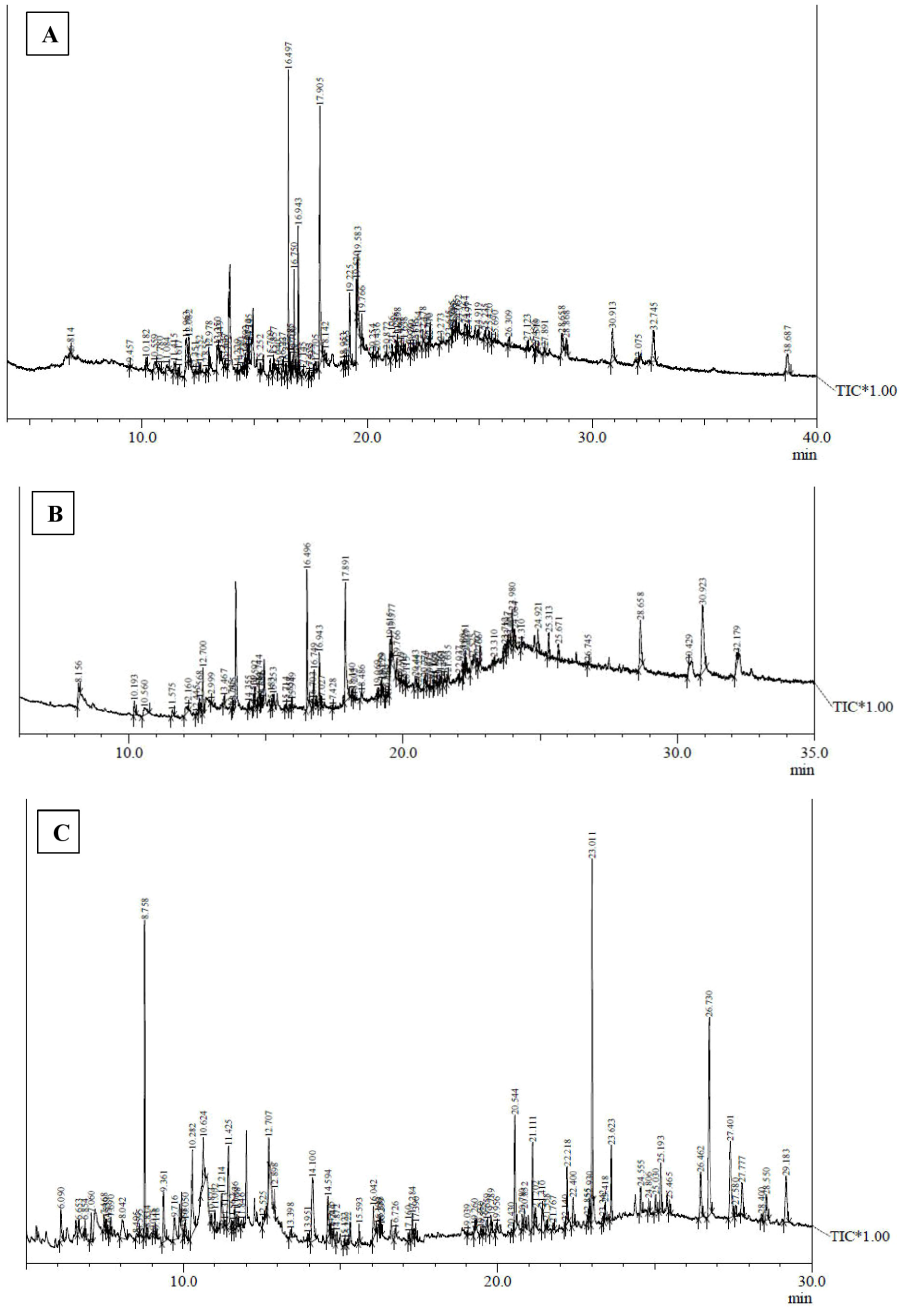
GC-MS chromatograms of methanolic extracts of **(A)** *in vivo* grown leaf tissue, **(B)** *in vitro* grown leaf tissue, and **(C)** leaf derived callus of *D. purpurea* L.

Metabolite profiling of methanolic extracts of field grown leaf, *in vitro* raised leaf and leaf-callus of *D. purpurea* were conducted by untargeted GC-MS technique. The phytocompounds in the extracts were identified by several parameters like retention time, fragment ions produced and comparison with the mass spectra of compounds indexed in the NIST library and the WILEY database. The retention time expressed in min, relative peak area percentage (%), molecular formula and molecular weight of compounds detected in each extract were listed in **Tables 6**–**8** and the respective chromatograms are presented in **Figure 3**. The several compounds found in each extract are known to possess multiple biological activities and belongs to diverse groups of secondary metabolites such as alkaloids, tannins, saponins, fatty acids, terpenoids, flavonoids, sterols etc. The *in vivo-* and *in vitro* grown leaf extract showed the presence of 86 and 77 phytocompounds respectively. Among the compounds detected in methanolic leaf extract grown *in vivo (* **Table 6**; **Figure 3A** *)* are: Palmitic acid (15.09%), Neophytadiene (11.49%), (E)-Phytol (5.62%), linolenic acid (4.84%), stigmasterin (3.85%), 1,11-Undecanediol (3.52%), Phytyl octadecenoate (3.06%), Linoleic acid (2.44%), alpha-tocopherol (2.14%), Gamma-undecanolactone (1.71%), loliolide (0.68%) and others. The methanolic extract of *in vitro* raised leaf tissue of *D. purpurea* displayed a number of phytoconstituents (**Table 7**; **Figure 3B**) such as stigmasterin (12.68%), palmitic acid (11.91%), neophytadiene (8.29%), methyl ester of linoleic acid (2.20%), 4-Vinylguaiacol (2.09%), Pyranone (2.04%), Ledol (1.43%), (-)-Cedrenol (0.50%), Glycidyl palmitate (0.28%) etc.

**Table 6:** List of phytocompounds identified in the methanolic extract of *in vivo* grown leaf tissue of *D. purpurea* L. by GC-MS.

S.No.	RT (min)	Peak Area %	Name of the Compound	Molecular Formula	Molecular Weight
1	6.814	0.21	2-trimethylsilyl-1,3-dithiane	C_7_H_16_S_2_Si	192
2	9.457	0.19	trimethyl-tetrahydronaphthalene	C_13_H_18_	174
3	10.182	0.55	5-methyl-2-isopropylphenol	C_10_H_14_O	150
4	10.559	0.77	4-vinylguaiacol	C_9_H_10_O_2_	150
5	10.780	0.17	1,1,5,6-tetramethylindane	C_13_H_18_	174
6	11.084	0.83	3-cyclohexene-1,1-dimethanol	C_8_H_14_O_2_	142
7	11.415	1.08	9-oxa-bicyclo [3.3.1] nonane-2,7-diol	C_8_H_14_O_3_	158
8	11.617	0.52	tetradecanolactone	C_14_H_26_O_2_	226
9	11.963	3.52	1,11-undecanediol	C_11_H_24_O_2_	188
10	12.082	1.71	gamma-undecanolactone	C_11_H_20_O_2_	184
11	12.351	0.22	glutaric acid, di(myrtenyl) ester	C_25_H_36_O_4_	400
12	12.552	0.44	succinic acid, tridec-2-yn-1-yl 2-methylpentyl ester	C_23_H_40_O_4_	380
13	12.855	0.23	4-butoxyaniline	C_10_H_15_NO	165
14	12.978	1.15	1-[(2z)-2-ethylidene-1-hydroxycyclohexyl] ethanone	C_10_H_16_O_2_	168
15	13.350	1.55	1,6-anhydro-beta-d-glucopyranose	C_6_H_10_O_5_	162
16	13.449	0.23	globulol	C_15_H_26_O	222
17	13.647	0.38	lauric acid	C_12_H_24_O_2_	200
18	13.792	0.09	2,2,4-trimethylpentanediol-1,3-diisobutyrate	C_16_H_30_O_4_	286
19	14.229	0.14	3-hydroxy-beta-damascone	C_13_H_20_O_2_	208
20	14.380	0.52	megastigmatrienone	C_13_H_18_O	190
21	14.592	0.59	dihydro methyl jasmonate	C_13_H_22_O_3_	226
22	14.650	0.16	3-oxo-alpha-ionol	C_13_H_20_O_2_	208
23	14.710	0.35	dihydroselarene	C_20_H_34_	274
24	14.745	0.29	beta-methylionone	C_14_H_22_O	206
25	15.252	0.47	1-heptadec-1-ynyl-cyclohexanol	C_23_H_42_O	334
26	15.709	1.33	2-hexylcinnamaldehyde	C_15_H_20_O	216
27	15.857	0.81	myristic acid	C_14_H_28_O_2_	228
28	16.040	0.08	3-phenylpropanal	C_9_H_10_O	134
29	16.227	0.68	loliolide	C_11_H_16_O_3_	196
30	16.372	0.39	isopropyl tetradecanoate	C_17_H_34_O_2_	270
31	16.497	11.49	neophytadiene	C_20_H_38_	278
32	16.585	0.61	6,10,14-trimethyl-2-pentadecanone	C_18_H_36_O	268
33	16.700	0.78	hexamethyl-pyranoindane	C_18_H_26_O	258
34	16.750	3.42	3,7,11,15-tetramethyl-2-hexadecen-1-ol	C_20_H_40_O	296
35	16.895	0.03	adipic acid, pentyl propyl ester	C_14_H_26_O_4_	258
36	16.943	5.62	(e)-phytol	C_20_H_40_O	296
37	17.145	0.40	eicosanoic acid, phenylmethyl ester	C_27_H_46_O_2_	402
38	17.423	0.25	hexadecanoic acid, methyl ester	C_17_H_34_O_2_	270
39	17.535	0.17	octadecyl chloride	C_18_H_37_Cl	288
40	17.705	0.56	2,6-dimethyltridecanenitrile	C_15_H_29_N	223
41	17.905	15.09	palmitic acid	C_16_H_32_O_2_	256
42	18.142	0.57	2-hexyl-1-decanol	C_16_H_34_O	242
43	18.953	0.20	cis-1-chloro-9-octadecene	C_18_H_35_Cl	286
44	19.065	0.25	2-methyltetracosane	C_25_H_52_	352
45	19.225	5.39	3,7,11,15-tetramethylhexadec-2-en-1-ol	C_20_H_40_O	296
46	19.520	2.44	linoleic acid	C_18_H_32_O_2_	280
47	19.583	4.84	linolenic acid	C_18_H_30_O_2_	278
48	19.766	1.71	stearic acid	C_18_H_36_O_2_	284
49	20.254	0.16	methyl 8-(3-octyl-2-oxiranyl) octanoate	C_19_H_36_O_3_	312
50	20.436	0.55	methyl-1-isopropyl-7-phenanthrene	C_18_H_18_	234
51	20.872	0.21	glycidyl palmitate	C_19_H_36_O_3_	312
52	21.106	0.78	9-octadecenoic acid, methyl ester	C_19_H_36_O_2_	296
53	21.296	0.60	n-eicosylcyclohexane	C_26_H_52_	364
54	21.358	0.72	delta-tridecalactone	C_13_H_24_O_2_	212
55	21.499	0.25	eicosanoic acid	C_20_H_40_O_2_	312
56	21.638	0.46	5-methyl-4-(prop-1-en-2-ylsulfanyl)-2h,3h-cyclopenta[a]naphthalen-1-one	C_17_H_16_OS	268
57	21.920	0.07	methyl 18-oxidanyloctadeca-9,12-dienoate, tms derivative	C_22_H_42_O_3_Si	382
58	21.960	0.31	11-tetradecen-1-ol acetate	C_16_H_30_O_2_	254
59	22.115	0.23	2-hydroxyethyl stearate	C_20_H_40_O_3_	328
60	22.264	0.30	heptadecyl octanoate	C_25_H_50_O_2_	382
61	22.478	0.33	2-hexyldecanol	C_16_H_34_O	242
62	22.700	0.49	propanoic acid, pentafluoro-, 1-phenyl-1,2-ethanediyl ester	C_14_H_8_F_10_O_4_	430
63	22.770	0.08	9-decenyl laurate	C_22_H_42_O_2_	338
64	23.273	0.16	dimethyl 2-(2-piperidinylidene) malonate	C_10_H_15_NO_4_	213
65	23.645	0.38	8s,14-cedrandiol	C_15_H_26_O_2_	238
66	23.795	0.30	octadecyl 2-ethylhexanoate	C_26_H_52_O_2_	396
67	23.845	0.17	linoleyl acetate	C_20_H_36_O_2_	308
68	23.975	0.18	stearyl monoglyceride	C_21_H_44_O_3_	344
69	24.062	0.39	methyl dehydroabietate	C_21_H_30_O_2_	314
70	24.364	1.00	cholest-14-en-3-ol	C_27_H_46_O	386
71	24.497	0.24	phytyl heptadecanoate	C_37_H_72_O_2_	548
72	24.919	0.23	squalene	C_30_H_50_	410
73	25.235	0.40	alpha-tocospiro b	C_29_H_50_O_4_	462
74	25.420	1.25	alpha-tocospiro a	C_29_H_50_O_4_	462
75	25.690	0.39	4-methylcyclohexanone semicarbazone	C_8_H_15_N_3_O	169
76	26.309	0.60	2-octyl-3-pentadecyloxirane	C_25_H_50_O	366
77	27.123	0.33	retinol	C_20_H_30_O	286
78	27.450	0.11	3-bromo-1-propanol, tms derivative	C_6_H_15_BrOSi	210
79	27.519	0.47	5,5-diethylpentadecane	C_19_H_40_	268
80	27.891	0.29	22,23-dihydroergosterol	C_28_H_46_O	398
81	28.658	2.14	d-alpha-tocopherol	C_29_H_50_O_2_	430
82	28.868	1.07	phytyl stearate	C_38_H_74_O_2_	562
83	30.913	3.85	stigmasterin	C_29_H_48_O	412
84	32.075	0.05	dihydropleurotin	C_21_H_24_O_5_	356
85	32.745	3.93	(e)-3,7,11,15-tetramethylhexadec-2-en-1-yl decanoate	C_30_H_58_O_2_	450
86	38.687	3.06	phytyl octadecanoate	C_38_H_74_O_2_	562

**Table 7:** List of phytocompounds identified in the methanolic extract of *in vitro* grown leaf tissue of *D. purpurea* L. by GC-MS.

S.No.	RT (min)	Peak Area %	Name of the Compound	Molecular Formula	Molecular Weight
1	8.156	2.04	pyranone	C_6_H_8_O_4_	144
2	10.193	1.41	5-methyl-2-isopropylphenol	C_10_H_14_O	150
3	10.560	2.09	4-vinylguaiacol	C_9_H_10_O_2_	150
4	11.575	0.63	2-[2-(alpha-hydroxybenzyl) propylidene]-1,3-oxathiane	C_14_H_18_O_2_S	250
5	12.160	1.71	2-propanone, 2-propenylhydrazone	C_6_H_12_N_2_	112
6	12.445	0.17	o-tert-butylphenol	C_10_H_14_O	150
7	12.568	1.43	ledol	C_15_H_26_O	222
8	12.700	2.79	2-tridecynyl 2,6-difluorobenzoate	C_20_H_26_F_2_O_2_	336
9	12.999	0.14	7-epi-sesquithujene	C_15_H_24_	204
10	13.467	0.50	1-hydroxy-6-(3-isopropenyl-cycloprop-1-enyl)-6-methyl-heptan-2-one	C_14_H_22_O_2_	222
11	13.745	0.10	n,n-di(2-cyclohexen-1-yl)amine	C_12_H_19_N	177
12	13.795	0.22	2-propyltetrahydrofuran	C_7_H_14_O	114
13	14.355	0.55	2-bornyl valerate	C_15_H_26_O_2_	238
14	14.539	0.10	(-)-alpha-terpineol	C_10_H_18_O	154
15	14.592	1.71	dihydro methyl jasmonate	C_13_H_22_O_3_	226
16	14.744	1.62	beta-methylionone	C_14_H_22_O	206
17	14.790	0.13	4-(1,1,3,3-tetramethylbutyl) phenol	C_14_H_22_O	206
18	14.836	0.27	1-(4-isopropylphenyl)-2-methylpropyl acetate	C_15_H_22_O_2_	234
19	15.183	0.22	1,3-dimethyladamantane	C_12_H_20_	164
20	15.253	0.85	1-heptadec-1-ynyl-cyclohexanol	C_23_H_42_O	334
21	15.714	0.36	1-isobutyl-4-(1-methyl-2-propenyl) benzene	C_14_H_20_	188
22	15.858	0.38	n-tridecoic acid	C_13_H_26_O_2_	214
23	15.949	0.50	(-)-cedrenol	C_15_H_26_O	222
24	16.496	8.29	neophytadiene	C_20_H_38_	278
25	16.703	0.45	hexamethyl-pyranoindane	C_18_H_26_O	258
26	16.749	2.20	3,7,11,15-tetramethyl-2-hexadecen-1-ol	C_20_H_40_O	296
27	16.943	3.00	(e)-phytol	C_20_H_40_O	296
28	17.027	0.19	decanol	C_10_H_22_O	158
29	17.428	0.24	octadecanoic acid, methyl ester	C_19_H_38_O_2_	298
30	17.891	11.91	palmitic acid	C_16_H_32_O_2_	256
31	18.140	0.62	3-hydroxypropyl palmitate, tms derivative	C_22_H_46_O_3_Si	386
32	18.202	0.17	glutaric acid, dodec-2-en-1-yl tetradecyl ester	C_31_H_58_O_4_	494
33	18.486	0.35	1,1,2,3,3-pentachloropropane	C_3_H_3_Cl_5_	214
34	19.069	0.61	heneicosane	C_21_H_44_	296
35	19.199	0.45	cis-1-chloro-9-octadecene	C_18_H_35_Cl	286
36	19.229	0.49	3,7,11,15-tetramethylhexadec-2-en-1-ol	C_20_H_40_O	296
37	19.342	0.19	arachidic acid methyl ester	C_21_H_42_O_2_	326
38	19.449	0.51	20-(5-methyltetrahydro-2h-pyran-2yl) pregnane	C_27_H_46_O	386
39	19.515	2.20	linoleic acid, methyl ester	C_19_H_34_O_2_	294
40	19.577	2.05	linolenic acid	C_18_H_30_O_2_	278
41	19.766	1.81	stearic acid	C_18_H_36_O_2_	284
42	19.903	0.32	3,5-di-t-butyl-4-hydroxyanisole	C_15_H_24_O_2_	236
43	20.010	0.53	3,5-dimethoxycyclohexanol	C_8_H_16_O_3_	160
44	20.115	0.09	7,7-dimethoxyheptanal	C_9_H_18_O_3_	174
45	20.443	1.18	methyl-1-isopropyl-7-phenanthrene	C_18_H_18_	234
46	20.555	0.16	14-heptacosanone	C_27_H_54_O	394
47	20.774	0.66	dimethylaminoethyl palmitate	C_20_H_41_NO_2_	327
48	20.874	0.28	glycidyl palmitate	C_19_H_36_O_3_	312
49	20.967	0.26	2-pyrrolidoneacetamide	C_6_H_10_N_2_O_2_	142
50	21.109	0.31	fumaric acid, dec-4-enyl tridecyl ester	C_27_H_48_O_4_	436
51	21.167	0.54	1-benzothiophene-3-carboxylic acid	C_9_H_6_O_2_S	178
52	21.295	0.30	octyl octanoate	C_16_H_32_O_2_	256
53	21.361	0.39	phytyl decanoate	C_30_H_58_O_2_	450
54	21.490	0.19	chloromethyl 6-chloroheptanoate	C_8_H_14_Cl_2_O_2_	212
55	21.635	0.47	neoergosterol	C_27_H_40_O	380
56	22.037	0.18	2-(1-undecyl) benzene-1,3-dicarbonitrile	C_23_H_34_O_2_	342
57	22.180	1.10	dimethylaminoethyl oleate	C_22_H_43_NO_2_	353
58	22.261	1.21	n-methyl-n,n-bis(3-aminopropyl)amine	C_7_H_19_N_3_	145
59	22.307	0.67	2-(heptyloxy)-4,6-dimethyl-1,3,2-dioxaborinane	C_12_H_25_BO_3_	228
60	22.525	1.10	ammodendrine	C_12_H_20_N_2_O	208
61	22.707	0.55	nonanoic acid, hexyl ester	C_15_H_30_O_2_	242
62	22.766	0.17	pentadecyl nonanoate	C_24_H_48_O_2_	368
63	23.310	0.32	dehydroabietic acid, methyl ester	C_22_H_32_O_2_	328
64	23.712	1.19	1-bromotriacontane	C_30_H_61_Br	500
65	23.797	0.63	4-methyloctadecanoic acid	C_19_H_38_O_2_	298
66	23.904	0.55	1-chloro-1-(3,3-diethoxy-1-propynyl)-2,2,3,3-tetramethylcyclopropane	C_14_H_23_ClO_2_	258
67	23.980	2.31	13-methylheptacosane	C_28_H_58_	394
68	24.064	1.57	dehydroabietic acid	C_21_H_30_O_2_	314
69	24.310	0.11	2-chloro-4-dodecylphenol	C_18_H_29_ClO	296
70	24.921	1.89	squalene	C_30_H_50_	410
71	25.313	2.32	tetrapentacontane	C_54_H_110_	758
72	25.671	1.22	tetracosane	C_24_H_50_	338
73	26.745	0.16	octyl 12-hydroxyoctadecanoate	C_26_H_52_O_3_	412
74	28.658	6.52	alpha-tocopherol	C_29_H_50_O_2_	430
75	30.429	0.53	(-)-beta-sitosterol	C_29_H_50_O	414
76	30.923	12.68	stigmasterin	C_29_H_48_O	412
77	32.179	1.68	gamma sitosterol	C_29_H_50_O	414

**Table 8:** List of phytocompounds identified in the methanolic extract of leaf derived callus of *D. purpurea* L. by GC-MS.

S.No.	RT (min)	Peak Area %	Name of the Compound	Molecular Formula	Molecular Weight
1	6.090	1.26	pyranone	C_6_H_8_O_4_	144
2	6.653	0.37	n-ethyl-n-methyl-2-propen-1-amine	C_6_H_13_N	99
3	6.854	0.28	decanal	C_10_H_20_O	156
4	7.060	0.80	prenyl isobutyrate	C_9_H_16_O_2_	156
5	7.468	0.43	linalool acetate	C_12_H_20_O_2_	196
6	7.539	0.30	geraniol	C_10_H_18_O	154
7	7.615	0.16	1-monoacetin	C_5_H_10_O_4_	134
8	7.690	0.43	beta-cyclocitral	C_10_H_16_O	152
9	8.042	1.82	1-ethylcyclohexanol	C_8_H_16_O	128
10	8.495	0.07	4-vinylguaiacol	C_9_H_10_O_2_	150
11	8.595	0.20	4-deoxypyridoxine	C_8_H_11_NO_2_	153
12	8.758	7.50	1,2-diacetylglycerol	C_7_H_12_O_5_	176
13	8.834	0.22	pulegone	C_10_H_16_O	152
14	9.025	0.08	eugenol	C_10_H_12_O_2_	164
15	9.118	0.08	2-methyl undecanal	C_12_H_24_O	184
16	9.361	1.89	9-oxabicyclo [3.3.1] nonane-2,6-diol	C_8_H_14_O_3_	158
17	9.716	1.61	4-ethylcatechol	C_8_H_10_O_2_	138
18	9.998	0.15	1,1-dimethyl-3-methylene-2-vinylcyclohexane	C_11_H_18_	150
19	10.050	0.62	3-octadecenal	C_18_H_34_O	266
20	10.282	5.30	cinnamic acid	C_9_H_8_O_2_	148
21	10.624	2.16	4-hydroxy-2,4,5-trimethyl-2,5-cyclohexadien-1-one	C_9_H_12_O_2_	152
22	10.873	0.24	ethyl propylphosphonofluoridate	C_5_H_12_FO_2_P	154
23	11.002	0.61	5-hydroxy-4,7,7-trimethylbicyclo [2.2.1] heptan-2-one	C_10_H_16_O_2_	168
24	11.214	1.54	6-dodecanol	C_12_H_26_O	186
25	11.313	0.18	jasmone	C_11_H_16_O	164
26	11.425	4.15	2-methylenecyclohexanol	C_7_H_12_O	112
27	11.562	0.37	delta.1,9-10-methyl-2-octalone	C_11_H_16_O	164
28	11.636	0.65	decyl formate	C_11_H_22_O_2_	186
29	11.708	0.37	lauric acid	C_12_H_24_O_2_	200
30	11.846	0.31	ethyl 2,3-nonadienoate	C_11_H_18_O_2_	182
31	12.525	0.29	tridecanedial	C_13_H_24_O_2_	212
32	12.707	1.39	dihydro methyl jasmonate	C_13_H_22_O_3_	226
33	12.898	0.43	1-(4-isopropylphenyl)-2-methylpropyl acetate	C_15_H_22_O_2_	234
34	13.398	0.22	stearyl acetate	C_20_H_40_O_2_	312
35	13.951	0.13	tridecanoic acid	C_13_H_26_O_2_	214
36	14.100	4.18	3-bornanone oxime	C_10_H_17_NO	167
37	14.594	1.72	4-(2,2,6-trimethylbicyclo [4.1.0] hept-1-yl)-2-butanone	C_14_H_24_O	208
38	14.675	0.08	neophytadiene	C_20_H_38_	278
39	14.744	0.12	6,10,14-trimethyl-2-pentadecanone	C_18_H_36_O	268
40	14.872	0.35	hexamethyl-pyranoindane	C_18_H_26_O	258
41	15.122	0.15	4,8,13-duvatriene-1,3-diol	C_20_H_34_O_2_	306
42	15.222	0.16	methyl 4-o-benzyl-alpha-l-rhamnopyranoside	C_14_H_20_O_5_	268
43	15.593	0.45	methyl palmitate	C_17_H_34_O_2_	270
44	16.042	1.37	n-hexadecanoic acid	C_16_H_32_O_2_	256
45	16.190	0.23	trehalose	C_12_H_22_O_11_	342
46	16.255	0.09	trans-10-phenyl-2-decalone	C_16_H_20_O	228
47	16.299	0.11	methyl pentacosanoate	C_26_H_52_O_2_	396
48	16.726	0.32	octyl hexopyranoside	C_14_H_28_O_6_	292
49	17.160	0.23	1-nonadecanol	C_19_H_40_O	284
50	17.284	0.70	methyl elaidate	C_19_H_36_O_2_	296
51	17.390	0.35	(e)-phytol	C_20_H_40_O	296
52	19.039	0.11	heptadecyl bromide	C_17_H_35_Br	318
53	19.260	0.23	oleic anhydride	C_36_H_66_O_3_	546
54	19.460	0.14	n-tetradecylcyclohexane	C_20_H_40_	280
55	19.525	0.17	methyl dehydroabietate	C_21_H_30_O_2_	314
56	19.659	0.30	5,7-dihydroxyflavone	C_15_H_10_O_4_	254
57	19.789	0.57	alpha-monostearin	C_21_H_42_O_4_	358
58	19.956	0.23	podocarpa-8,11,13-trien-3-one	C_17_H_22_O	242
59	20.430	0.06	3-(3’,4’-dimethoxyphenyl) coumarin	C_17_H_14_O_4_	282
60	20.544	3.62	3-hydroxy-5-methoxyflavone	C_16_H_12_O_4_	268
61	20.780	0.42	1,5-dimethoxyanthraquinone	C_16_H_12_O_4_	268
62	20.852	0.55	2-monopalmitin	C_19_H_38_O_4_	330
63	21.111	2.60	1,5-dimethoxyanthra-9,10-quinone	C_16_H_12_O_4_	268
64	21.202	0.70	4,22-cholestadien-3-one	C_27_H_42_O	382
65	21.410	0.25	epi-allogibberinic acid	C_18_H_20_O_3_	284
66	21.558	0.22	2-hexyldecanol	C_16_H_34_O	242
67	21.767	0.21	3-methylcholanthren-2-ol	C_21_H_16_O	284
68	22.140	0.15	1-eicosene	C_20_H_40_	280
69	22.218	1.61	1-octacosanol	C_28_H_58_O	410
70	22.400	1.05	3-methoxyestra-1(10),2,4,6,8-pentaen-17-ol	C_19_H_22_O_2_	282
71	22.855	0.22	1-nonacosene	C_29_H_58_	406
72	22.930	0.54	methyl tetratriacontyl ether	C_35_H_72_O	508
73	23.011	9.06	1-tetratriacontanol, heptafluorobutyrate	C_38_H_69_F_7_O_2_	690
74	23.342	0.23	2-methylhexacosane	C_27_H_56_	380
75	23.418	0.66	3,17,20,21-tetrahydroxypregnan-11-one	C_21_H_34_O_5_	366
76	23.623	2.08	heneicosyl trifluoroacetate	C_23_H_43_F_3_O_2_	408
77	24.555	0.93	stigmasta-4,7,22-trien-3-beta-ol	C_29_H_46_O	410
78	24.806	0.38	beta-amyrin	C_30_H_50_O	426
79	25.030	0.62	22,23-dihydroergosterol	C_28_H_46_O	398
80	25.193	1.78	methyl commate a	C_32_H_52_O_4_	500
81	25.465	0.43	5-cholesten-3-beta-ol	C_27_H_46_O	386
82	26.462	2.24	campesterol	C_28_H_48_O	400
83	26.730	9.30	stigmasterol	C_29_H_48_O	412
84	27.401	4.83	gamma sitosterol	C_29_H_50_O	414
85	27.580	0.51	isofucosterol	C_29_H_48_O	412
86	27.777	1.60	7,22-ergostadienone	C_28_H_44_O	396
87	28.400	0.25	cycloartenol	C_30_H_50_O	426
88	28.550	1.30	3,5-cholestadien-7-one	C_27_H_42_O	382
89	29.183	2.58	24-methylcycloartenol	C_31_H_52_O	440

**Figure 4:**
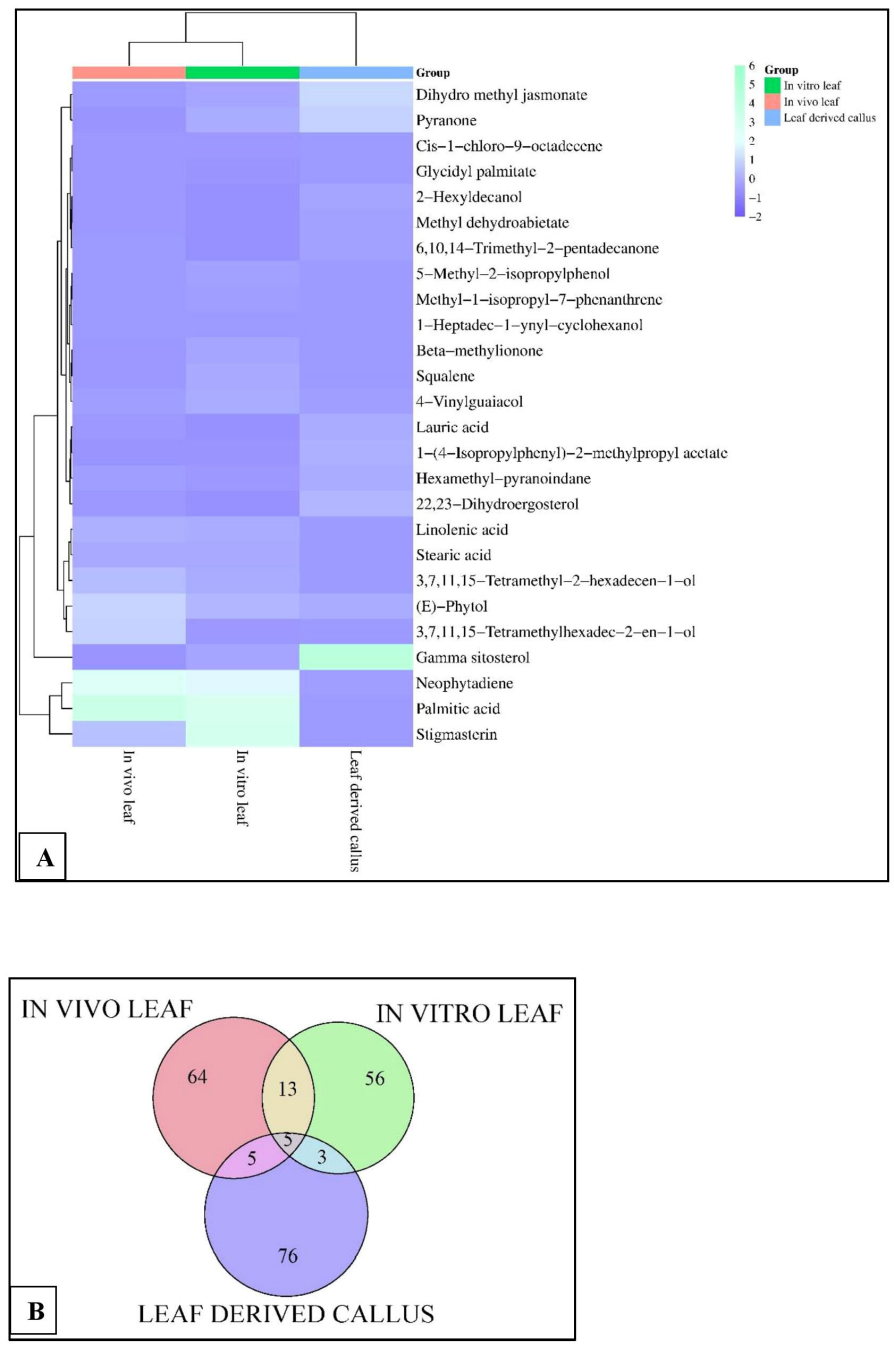
**(A)** Heatmap analysis, and **(B)** Venn diagram displaying the relative abundance of important phytocompounds in the *in vivo*-, *in vitro-* grown leaf and callus samples of *D. purpurea* L. detected by GC-MS.

The GC-MS profiling of *D. purpurea* callus extract revealed the presence of 89 bioactive compounds (**Table 8**; **Figure 3C**). the major bioactives identified by GC-MS analysis were Stigmasterol (9.30%), 1-Tetratriacontanol heptafluorobutyrate (9.06%), 1,2-Diacetylglycerol (7.50%), Cinnamic acid (5.30%), gamma sitosterol (4.83%), 3-bornanone oxime (4.18%), 3-Hydroxy-5-methoxyflavone (3.62%), Campesterol (2.24%), beta-Amyrin (0.38%), Lauric acid (0.37%) etc. The heatmap and the Venn diagram illustrated in the **Figure 4** indicated the common phytocompounds present in all the three or two samples examined. In total, 5 metabolites had been found to be present in all the three extracts, these were 4-Vinylguaiacol, dihydro methyl jasmonate, Neophytadiene, hexamethyl-pyranoindane, (E)-phytol; whereas 13 bioactives were found to be common in both *in vivo-* and *in vitro* derived leaf tissue. There are 5 secondary metabolites present in field grown leaf and leaf derived callus, while only 3 phytocompounds were common in leaf-callus and *in vitro* raised leaf tissue.

### 3.4 LC-MS based metabolites profiling

**Figure 5:**
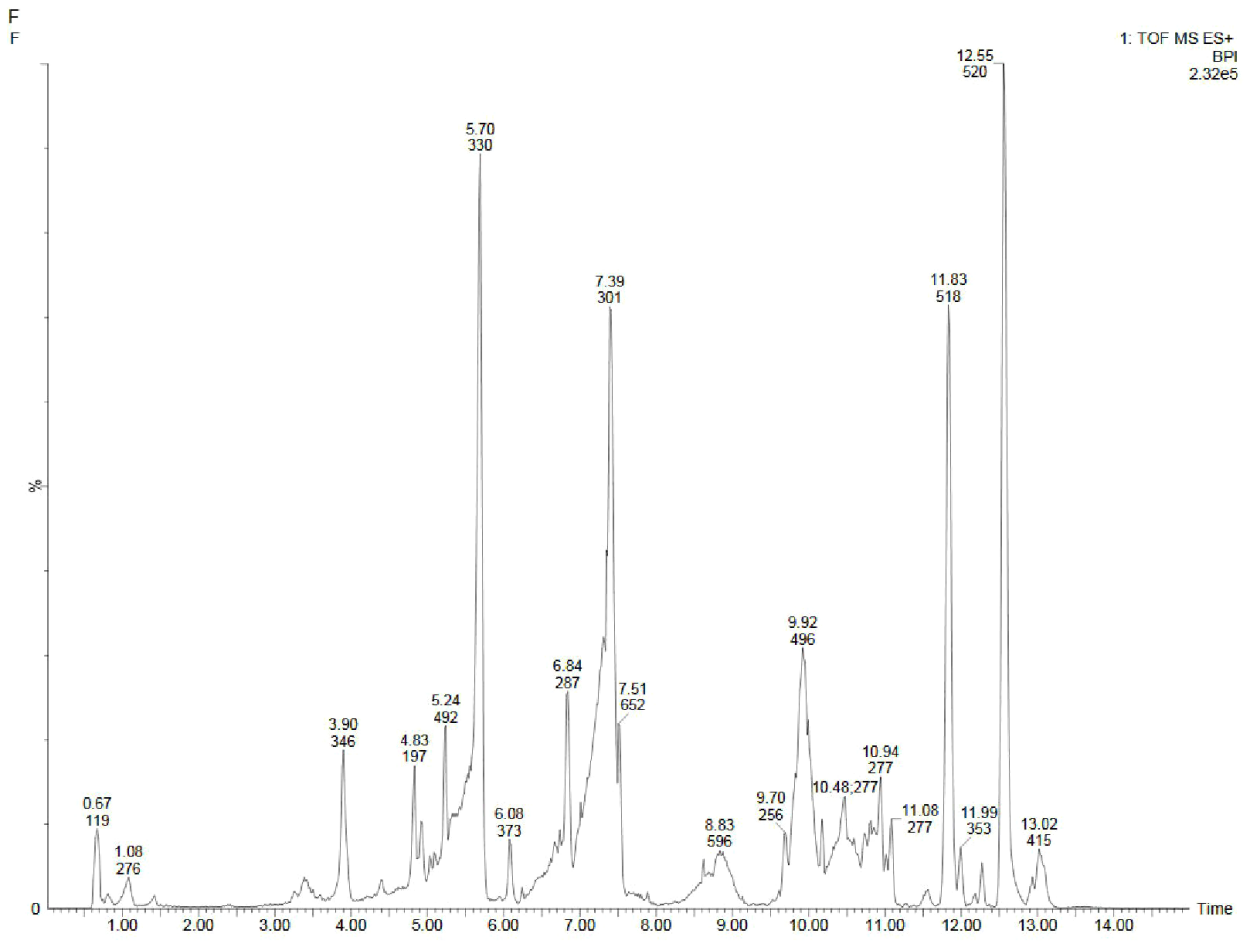
UPLC-ESI-QTOF-MS chromatogram of methanolic leaf extract of the *in vivo* grown *D. purpurea* L. in positive ionization mode.

**Figure 6:**
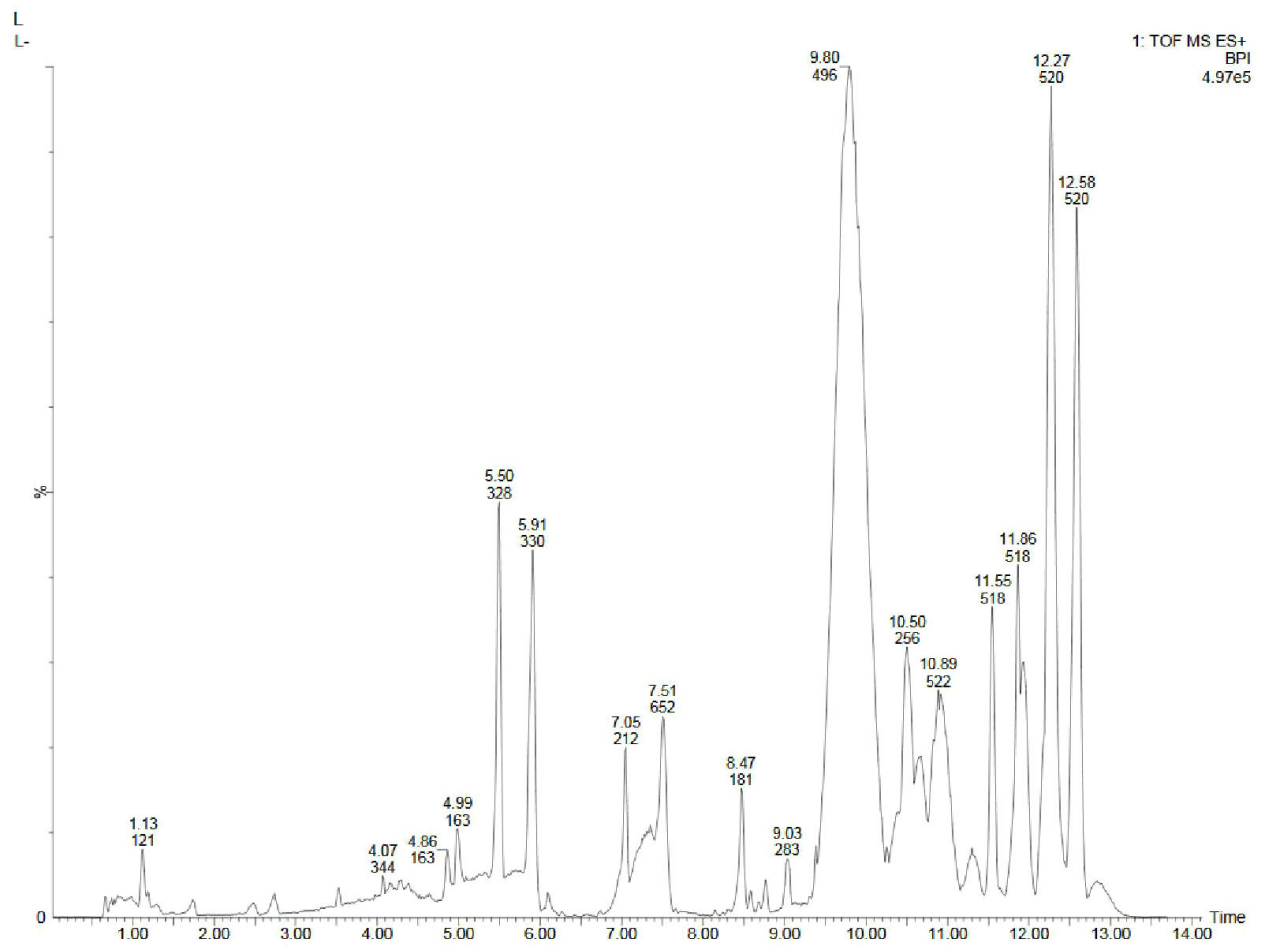
UPLC-ESI-QTOF-MS chromatogram of methanolic leaf extract of the *in vitro* grown *D. purpurea* L. in positive ionization mode.

**Figure 7:**
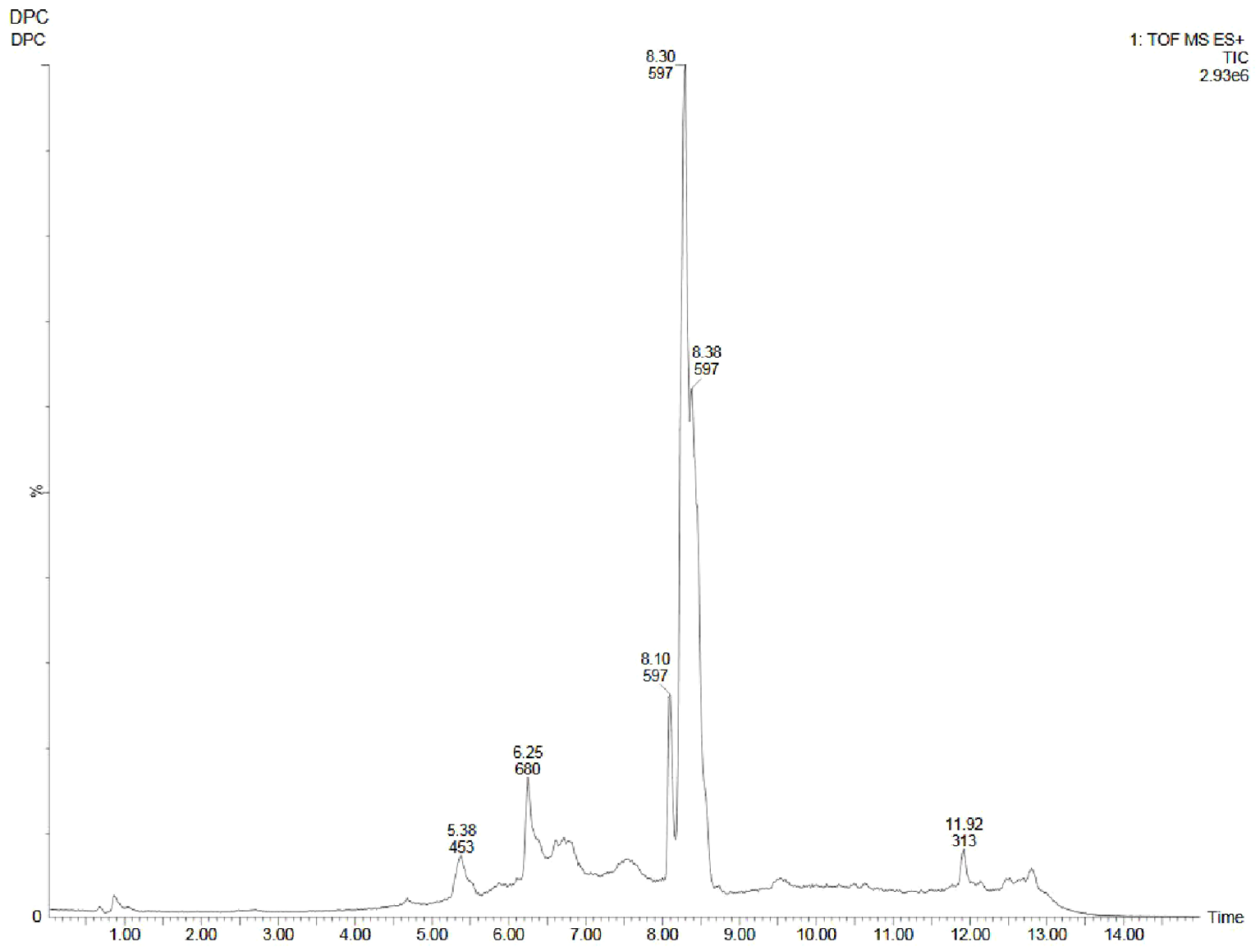
UPLC-ESI-QTOF-MS chromatogram of methanolic callus extract of *D. purpurea* L. in positive ionization mode.

**Figure 8:**
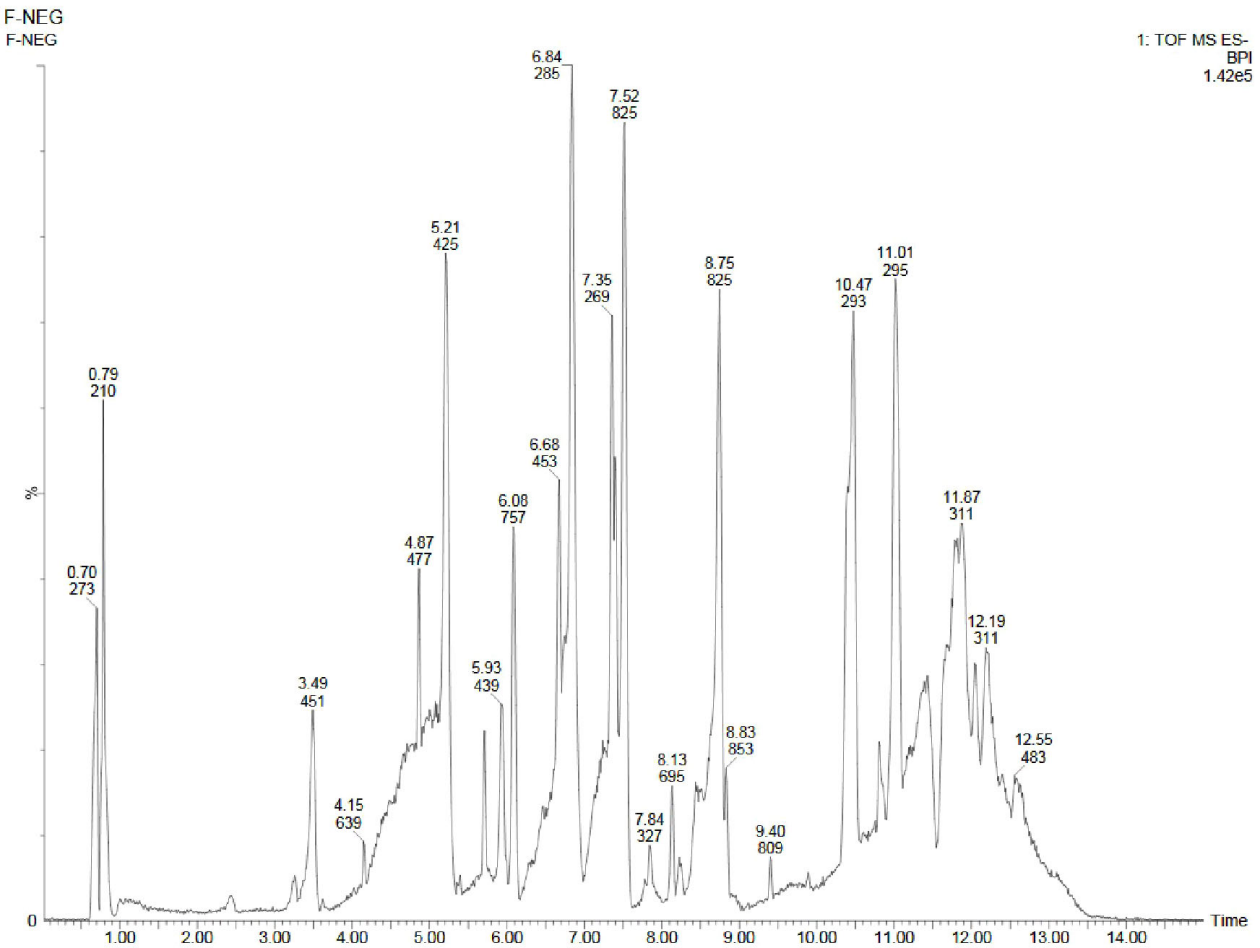
UPLC-ESI-QTOF-MS chromatogram of methanolic leaf extract of the *in vivo* grown *D. purpurea* L. in negative ionization mode.

**Figure 9:**
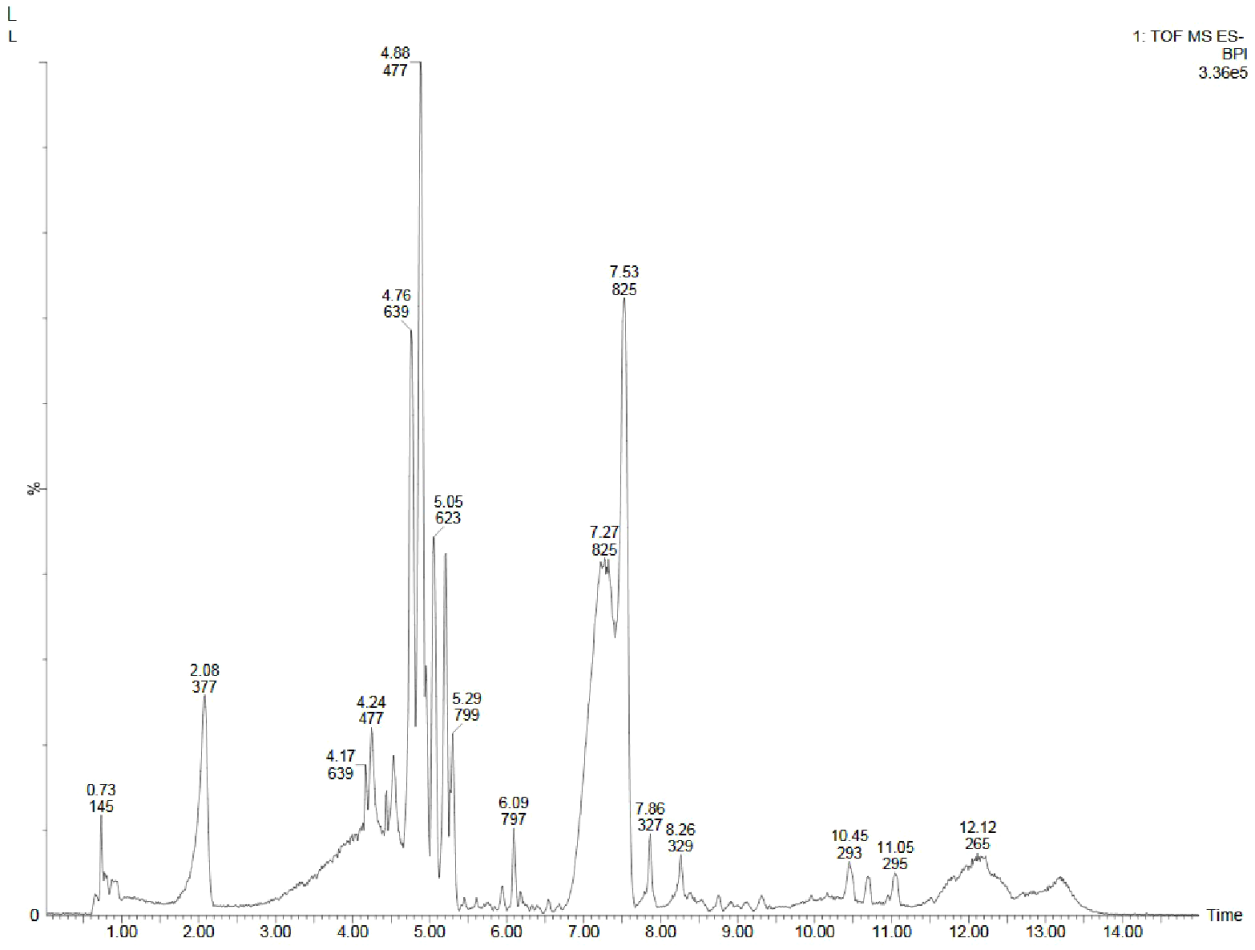
UPLC-ESI-QTOF-MS chromatogram of methanolic leaf extract of the *in vitro* grown *D. purpurea* L. in negative ionization mode.

**Figure 10:**
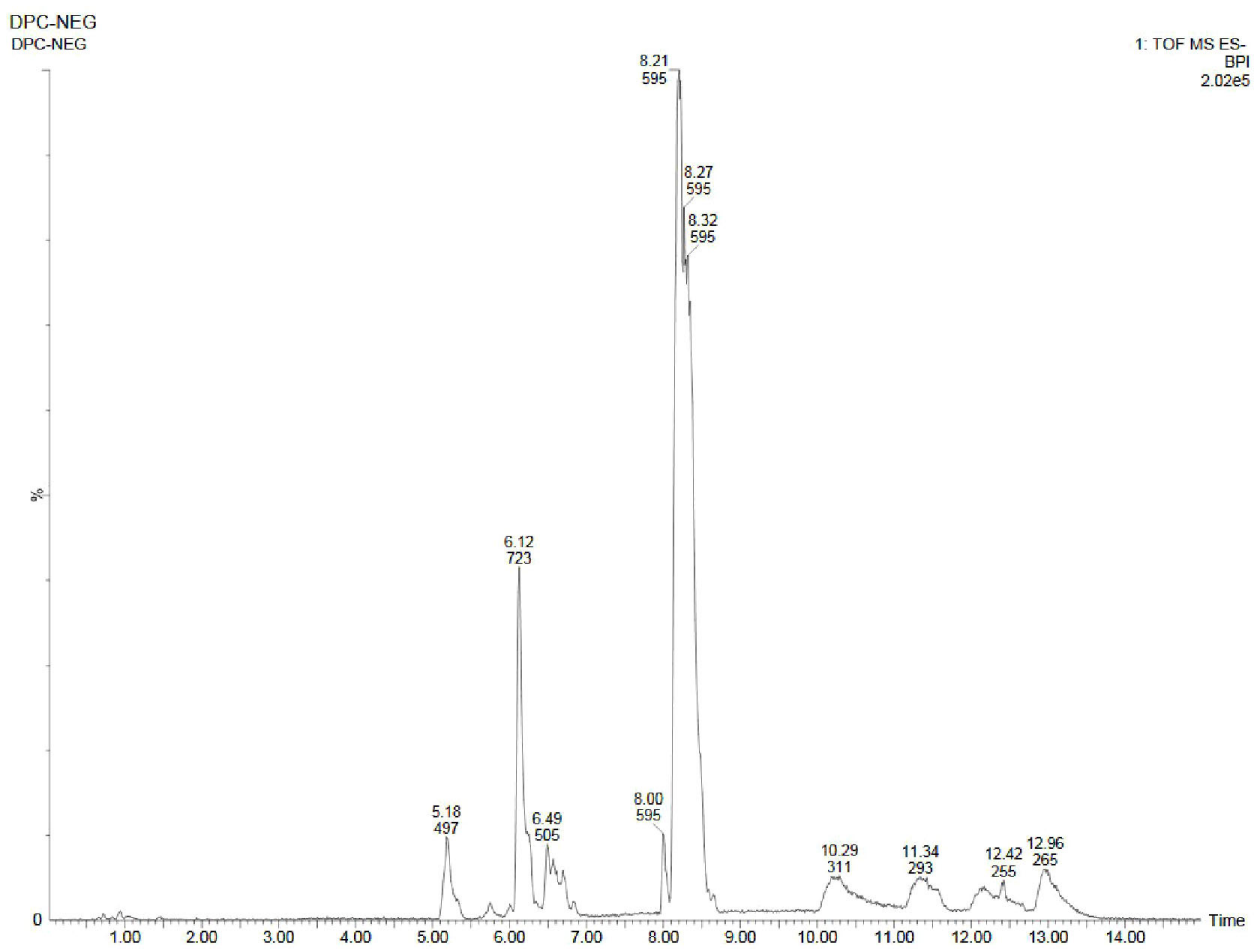
UPLC-ESI-QTOF-MS chromatogram of methanolic callus extract of *D. purpurea* L. in negative ionization mode.

Using UPLC-ESI-QTOF-MS method, the phytochemicals of field- and laboratory grown leaf and leaf-callus of *D. purpurea* were investigated in both the positive and negative ionization modes. The base peak chromatograms (positive and negative ion modes) of each sample are presented in **Figures 5**–**10** with the retention time and measured m/z of the compounds. The phytocompounds were identified by comparing the [M+H]^+^, [M+Na]^+^ and [M+H-H_2_O]^+^ protonated molecule (for positive mode) and [M-H]^-^ de-protonated molecule (for negative mode) and their fragmentation in MS/MS spectra with the data recorded in the HMDB and METLIN databases. The identified phytocompounds were tabulated in **Tables 9**–**14** with their retention time, measured m/z, exact m/z, molecular formula and product ions (ms/ms).

**Table 9:** Identification of phytocompounds in the methanolic leaf extract of *in vivo* grown *D. purpurea* L. by UPLC-ESI-QTOF-MS (positive ion mode) analysis.

S.No.	Rt (min)	Measured m/z	Exact m/z	Name of the compound	Ion	Formula	Product ions (m/z)	Category
1	0.67	119	118.9975	Mesoxalic acid	M+H	C_3_H_2_O_5_	19.01, 31.02,44.99,56.99,75.01	Carboxylic acids
2	1.08	276	275.2375	Methyl linolenate	M+H-H2O	C_19_H_32_O_2_	67.05, 79.05, 81.06, 93.06	Fatty acyls
3	3.9	346	346.3104	2,4,12-Octadecatrienoic acidpiperidide	M+H	C_23_H_39_NO	55.05, 67.05, 69.06, 79.05, 84.08, 86.09, 91.05	Piperidines
4	4.83	197	197.0057	Dehydroascorbic acid	M+Na	C_6_H_6_O_6_	43.02, 61.03, 115, 139,157.01, 175.02	Lactones
5	5.24	492	491.3859	Lupeol acetate	M+Na	C_32_H_52_O_2_	409.38, 427.39, 469.40	Triterpenoids
6	5.7	330	330.073	Glutathione	M+Na	C_10_H_17_N_3_O_6_S	76.05, 84.08, 129.99	Peptides
7	6.08	373	372.9989	Apigenin-7-sulfate	M+Na	C_15_H_10_O_8_S	119.05, 123.04, 197.06, 232.97, 234.99, 351.01	Flavonoids
8	6.84	287	287.055	Luteolin	M+H	C_15_H_10_O_6_	68.99, 89.03, 117.03,135.04,161.02, 241.04, 287.05	Flavonoids
9	7.39	301	301.0707	Kaempferid	M+H	C_16_H_12_O_6_	283.06, 285.03, 301.07	Flavonoids
10	7.51	652	651.4959	Diglyceride	M+Na	C_40_H_68_O_5_	261.22, 277.25, 335.25, 353.26, 611.50	Diacylglycerol
11	8.83	596	596.1524	Cyanidin 3-galactoside p-coumaric acid ester	M+H	C_30_H_27_O_13_	585.32, 596.15, 603.33	Flavonoids
12	9.7	256	257.2475	Octyl octanoate	M+H	C_16_H_32_O_2_	43.05, 55.05, 57.06, 95.08, 127.11	Fatty acyls
13	9.92	496	495.3809	alpha-Tocopherol acetate	M+Na	C_31_H_52_O_3_	57.06, 71.08, 147.08, 165.09, 207.10, 413.37	Vitamin E
14	10.48	277	277.0319	6-Phosphogluconic acid	M+H	C_6_H_13_O_10_P	43.01, 56.99, 59.01,61.02, 73.02, 75.01, 98.98, 105.01, 161.04	Monosaccharide phosphates
15	10.94	277	275.1981	Linalyl hexanoate	M+Na	C_16_H_28_O_2_	41.03, 43.05, 53.03, 55.05, 57.06, 67.05, 69.06, 99.08	Monoterpenoid
16	11.08	277	278.2454	Hexadecanamide	M+Na	C_16_H_33_NO	211.24, 221.22, 238.25, 239.23, 256.26	Fatty acyls
17	11.83	518	517.1341	Dicaffeoylquinic acid	M+H	C_25_H_24_O_12_	117.02, 120.98,144.38, 163.03	Quinic acids and derivatives
18	11.99	353	353.4142	Isopentacosane	M+H	C_25_H_52_	43.05, 57.07, 225.25, 239.27, 253.28, 295.33	Saturated hydrocarbon
19	12.55	520	521.3109	Digoxigenin monodigitoxoside	M+H	C_29_H_44_O_8_	137.07, 373.23, 391.24, 503.30	Steroid saponin
20	13.02	415	415.2455	20, 22-Dihydrodigoxigenin	M+Na	C_23_H_36_O_5_	357.24, 375.25, 393.26	Cardenolides

**Table 10:** Identification of phytocompounds in the methanolic leaf extract of *in vitro* grown *D. purpurea* L. by UPLC-ESI-QTOF-MS (positive ion mode) analysis.

S.No.	Rt (min)	Measured m/z	Exact m/z	Name of the compound	Ion	Formula	Product ions (m/z)	Category
1	1.13	121	121.0624	Hexenal	M+Na	C_6_H_10_O	81.07, 99.08	Carbonyl compound
2	4.07	344	343.0818	Rosmarinic acid	M+H-H2O	C_18_H_16_O_8_	123.04, 135.04, 163.03, 181.05, 199.306, 43.08	Polyphenol
3	4.86	163	163.0607	D-Mannose	M+H-H2O	C_6_H_12_O_6_	43.01, 45.03, 57.03, 61.02, 75.04, 85.02, 103.03, 181.07	Carbohydrates
4	4.99	163	163.1851	(Z)-1,5-Tridecadiene	M+H-H2O	C_13_H_24_	181.19	Unsaturated hydrocarbon
5	5.5	328	329.3414	1,21-Heneicosanediol	M+H	C_21_H_44_O_2_	311.33, 329.34	Fatty acyls
6	5.91	330	330.073	Glutathione	M+Na	C_10_H_17_N_3_O_6_S	76.05, 84.08, 129.99	Peptides
7	7.05	212	213.1485	Dihydrojasmonic acid	M+H	C_12_H_20_O_3_	41.03, 43.01, 55.01, 79.05, 81.06, 83.08	Fatty acyls
8	7.51	652	651.4959	Diglyceride	M+Na	C_40_H_68_O_5_	261.22, 277.25, 335.25, 353.26, 611.50	Diacylglycerol
9	8.47	181	181.1199	2-Heptyl acetate	M+Na	C_9_H_18_O_2_	41.03, 43.05, 55.05, 57.06, 69.06, 71.08	Carboxylic acids
10	9.03	283	283.0607	Luteolin 7-methyl ether	M+H-H2O	C_16_H_12_O_6_	301.07	Flavonoids
11	9.8	496	495.3809	alpha-Tocopherol acetate	M+Na	C_31_H_52_O_3_	57.06, 71.08, 147.08, 165.09, 207.10, 413.37	Vitamin E compound
12	10.5	256	257.2475	Octyl octanoate	M+H	C_16_H_32_O_2_	43.05, 55.05, 57.06, 95.08, 127.11	Fatty acyls
13	10.89	522	521.5656	5-Hexatriacontanone	M+H	C_36_H_72_O	503.55	Carbonyl compound
14	11.55	518	517.1341	Dicaffeoylquinic acid	M+H	C_25_H_24_O_12_	117.02, 120.98,144.38, 163.03	Quinic acids and derivatives
15	11.86	518	519.0928	Sagecoumarin	M+H-H2O	C_27_H_20_O_12_	123.04, 181.04, 311.05, 339.04	Coumarins and derivatives
16	12.27	520	521.3109	Digoxigenin monodigitoxoside	M+H	C_29_H_44_O_8_	137.07, 373.23, 391.24, 503.30	steroid saponin
17	12.58	520	521.0902	Methyl 4,6-di-O-galloyl-beta-D-glucopyranoside	M+Na	C_21_H_22_O_14_	153.01, 329.08, 373.07, 443.11, 499.10	Tannins

**Table 11:** Identification of phytocompounds in the methanolic callus extract of *D. purpurea* L. by UPLC-ESI-QTOF-MS (positive ion mode) analysis.

S.No.	Rt (min)	Measured m/z	Exact m/z	Name of the compound	Ion	Formula	Product ions (m/z)	Category
1	5.38	453	453.1391	Epicatechin 3-glucoside	M+H	C_21_H_24_O_11_	145.04, 291.08, 393.11, 435.12, 453.13	Flavonoids
2	6.25	680	681.3487	(15a,20R)-Dihydroxypregn-4-en-3-one 20-[glucosyl-(1->4)-6-acetyl-glucoside]	M+H-H2O	C_35_H_54_O_14_	315.23, 333.24, 519.29, 537.30	Steroids
3	8.1	597	597.145	Quercetin 3-glucoside 7-xyloside	M+H	C_26_H_28_O_16_	435.09, 597.14	Flavonoids
4	8.3	597	597.4126	stigmasterol 3-O-beta-D-glucoside	M+Na	C_35_H_58_O_6_	95.08, 97.10, 395.36, 413.37	Steroids
5	8.38	597	597.5241	Erythrodiol 3-decanoate	M+H	C_40_H_68_O_3_	275.23, 429.37, 443.38, 485.39	Triterpenoids
6	11.92	313	313.1071	3’,4’,5’-Trimethoxyflavone	M+H	C_18_H_16_O_5_	313.1	Flavonoids

**Table 12:** Identification of phytocompounds in the methanolic leaf extract of *in vivo* grown *D. purpurea* L. by UPLC-ESI-QTOF-MS (negative ion mode) analysis.

S.No.	Rt (min)	Measured m/z	Exact m/z	Name of the compound	Ion	Formula	Product ions (m/z)	Category
1	0.7	273	273.0768	3,3’,4’,7-Tetrahydroxyflavan	M-H	C_15_H_14_O_5_	121.02, 151.03, 273.07	Flavonoids
2	0.79	210	210.1267	(9z)-12-oxo-dodec-9-enoate	M-H	C_12_H_19_O_3_	167.14, 183.13, 193.12, 211.13	Fatty acyls
3	3.49	451	451.1246	Epicatechin 3-glucoside	M-H	C_21_H_24_O_11_	43.01, 109.02, 125.02, 289.07	Flavonoids
4	4.15	639	639.0992	Quercetin 3-(2-caffeoylglucuronoside)	M-H	C_30_H_24_O_16_	161.02, 179.03, 283.02, 301.03	Flavonoids
5	4.87	477	477.1039	Nepitrin	M-H	C_22_H_22_O_12_	299.01, 315.05	Flavonoids
6	5.21	425	425.3789	alpha-Amyrin	M-H	C_30_H_50_O	407.36, 425.37	Triterpenoids
7	5.93	439	439.3945	24-Methylcycloartenol	M-H	C_31_H_52_O	421.38, 439.39	Steroids
8	6.08	757	755.204	Kaempferol 3-coumaroyl-triglucoside	M-H	C_33_H_40_O_20_	431.09, 755.20	Flavonoids
9	6.68	453	454.3458	4alpha-carboxy-stigmasta-7,24(241)-dien-3beta-ol	M-H	C_30_H_40_O_3_	393.35, 411.36, 437.34	Steroids
10	6.84	285	285.0405	Luteolin	M-H	C_15_H_10_O_6_	285.03	Flavonoids
11	7.35	269	269.0455	Apigenin	M-H	C_15_H_10_O_5_	269.04	Flavonoids
12	7.52	825	825.6978	Triacylglycerol	M-H	C_53_H_94_O_6_	61.02, 253.21, 279.23, 319.26, 335.25	Triacylglycerol
13	7.84	327	327.3269	1,21-Heneicosanediol	M-H	C_21_H_44_O_2_	297.31, 309.31, 327.32	Fatty acyls
14	8.13	695	697.3441	(15a,20R)-Dihydroxypregn-4-en-3-one 20-[glucosyl-(1->4)-6-acetyl-glucoside]	M-H	C_35_H_54_O_14_	59.01, 313.21, 331.22	Steroids
15	8.75	825	827.1888	Luteolin 7-O-(2-apiosyl-4-glucosyl-6-malonyl)-glucoside	M-H	C_35_H_40_O_23_	59.01, 84.99, 103, 149.04, 285.03	Flavonoids
16	8.83	853	853.2785	Hydroxymethylbilane	M-H	C_40_H_46_N_4_O_17_	791.24, 835.26	Tetrapyrroles
17	9.4	809	809.3026	isoorientin 6-O-hexoside	M-H	C_42_H_50_O_16_	181.05, 337.12, 415.13, 541.20, 597.19, 641.22, 763.26	lignans
18	10.47	293	293.2122	2-Hydroxylinolenic acid	M-H	C_18_H_30_O_3_	293.21	Fatty acyls
19	11.01	295	295.2643	Nonadecenoic acid	M-H	C_19_H_36_O_2_	277.25, 295.26	Fatty acyls
20	11.87	311	311.2956	Ethyl stearate	M-H	C_20_H_40_O_2_	265.25	Fatty acyls
21	12.19	311	311.2017	Delta4,16-Pregnadiene-3,20-dione	M-H	C_21_H_28_O_2_	269.19, 295.17	Steroids
22	12.55	483	483.078	2,6-Digalloylglucose	M-H	C_20_H_20_O_14_	125.02, 169.01	Benzenoids

**Table 13:** Identification of phytocompounds in the methanolic leaf extract of *in vitro* grown *D. purpurea* L. by UPLC-ESI-QTOF-MS (negative ion mode) analysis.

S.No.	Rt (min)	Measured m/z	Exact m/z	Name of the compound	Ion	Formula	Product ions (m/z)	Category
1	0.73	145	145.0295	Coumarin	M-H	C_9_H_6_O_2_	101.03, 145.02	Coumarins and derivatives
2	2.08	377	376.0378	2-Hydroxypropyl glucosinolate	M-H	C_10_H_19_NO_10_S_2_	111.97, 195.97, 376.03	Carbohydrates
3	4.17	639	639.0992	Quercetin 3-(2-caffeoylglucuronoside)	M-H	C_30_H_24_O_16_	161.02, 179.03, 283.02, 301.03	Flavonoids
4	4.24	477	477.1039	Nepitrin	M-H	C_22_H_22_O_12_	299.01, 315.05	Flavonoids
5	4.76	639	639.1567	Rhamnetin 3-laminaribioside	M-H	C_28_H_32_O_17_	315.05, 639.15	Carboxylic acids
6	4.88	477	477.1402	4’-O-Methyl-(-)-epicatechin-5-O-β-glucuronide	M-H	C_23_H_26_O_11_	163.07, 283.09, 301.10	Flavonoids
7	5.05	623	623.1618	3,8-Diglucosyldiosmetin	M-H	C_28_H_32_O_16_	623.16	Flavonoids
8	5.29	799	799.6457	Campesterol 6’-hexadecanoylglucoside	M-H	C_50_H_88_O_7_	399.36, 543.40, 561.41, 799.64	Steroids
9	6.09	797	797.1418	Apigenin 7-[glucuronyl-(1->2)-glucuronide] 4’-glucuronide	M-H	C_33_H_34_O_23_	444.06	Flavonoids
10	7.27	825	827.1888	Luteolin 7-O-(2-apiosyl-4-glucosyl-6-malonyl)-glucoside	M-H	C_35_H_40_O_23_	59.01, 84.99, 103, 149.04, 285.03	Flavonoids
11	7.53	825	825.6978	Triacylglycerol	M-H	C_53_H_94_O_6_	61.02, 253.21, 279.23, 319.26, 335.25	Triacylglycerol
12	7.86	327	327.3269	1,21-Heneicosanediol	M-H	C_21_H_44_O_2_	297.31, 309.31, 327.32	Fatty acyls
13	8.26	329	329.2122	pregn-5-ene-3,20-dione-17-ol	M-H	C_21_H_30_O_3_	287.20, 329.21	Steroids
14	10.45	293	293.2122	2-Hydroxylinolenic acid	M-H	C_18_H_30_O_3_	293.21	Fatty acyls
15	11.05	295	295.2643	Nonadecenoic acid	M-H	C_19_H_36_O_2_	277.25, 295.26	Fatty acyls
16	12.12	265	264.952	2,3-Diphosphoglyceric acid	M-H	C_3_H_8_O_10_P_2_	78.95, 96.96	Carbohydrates

**Table 14:** Identification of phytocompounds in the methanolic callus extract of *D. purpurea* L. by UPLC-ESI-QTOF-MS (negative ion mode) analysis.

S.No.	Rt (min)	Measured m/z	Exact m/z	Name of the compound	Ion	Formula	Product ions (m/z)	Category
1	5.18	497	497.0937	Methyl 4,6-di-O-galloyl-β-D-glucopyranoside	M-H	C_21_H_22_O_14_	125.02, 169.01, 441.10	Tannins
2	6.12	723	723.1719	2’’,3’’-Di-O-p-coumaroylafzelin	M-H	C_39_H_32_O_14_	151.00, 723.17	Flavonoids
3	6.49	505	505.0988	Quercetin 3-(6’’-acetylglucoside)	M-H	C_23_H_22_O_13_	59.01, 301.03	Flavonoids
4	8	595	595.4579	Squamoxinone C	M-H	C_35_H_64_O_7_	577.44, 595.45	Fatty acyl
5	8.21	595	595.5096	Erythrodiol 3-decanoate	M-H	C_40_H_68_O_3_	595.5	Triterpenoids
6	8.27	595	595.1668	Cassiaside C	M-H	C_27_H_32_O_15_	59.01, 241.05, 271.06	Naphthopyrans
7	8.32	595	595.4215	Diacylglycerol	M-H	C_34_H_60_O_8_	195.17, 213.18, 307.19, 381.22, 451.30	Diacylglycerol
8	10.29	311	311.2017	Delta4,16-Pregnadiene-3,20-dione	M-H	C_21_H_28_O_2_	269.19, 295.17	Steroids
9	11.34	293	293.2122	2-Hydroxylinolenic acid	M-H	C_18_H_30_O_3_	293.21	Fatty acyl
10	12.42	255	255.233	Octyl octanoate	M-H	C_16_H_32_O_2_	125.09, 143.10, 255.23	Fatty acyl
11	12.96	265	264.952	2,3-Diphosphoglyceric acid	M-H	C_3_H_8_O_10_P_2_	78.95, 96.96	Carbohydrates

#### 3.4.1 Analyses in the positive-ion mode

The *in vivo* grown leaf sample contained a total of 20 metabolites in 40 min elution time, mostly of which were flavonoids and fatty acyl groups. The first eluted compound was mesoxalic acid with a mass of 119 m/z at 0.67 min (**Table 9**; **Figure 5**). Various flavonoids such as apigenin-7-sulfate (6.08 min), luteolin (6.84 min) and kaempferide (7.39 min) were identified with the mass of 372.99 m/z, 287.05 m/z and 301.07 m/z, respectively. Also, certain fatty acyls like methyl linolenate (1.08 min), octyl octanoate (9.70 min) and hexadecanamide (11.08 min) were detected having masses of 275.23 m/z, 257.24 m/z and 278.24 m/z, respectively. An important cardenolide (20,22-Dihydrodigoxigenin) was also found in *in vivo* leaf extract at 13.02 min with the mass of 415.24 m/z. The LC-MS analysis of *in vitro* leaf extract of *D. purpurea* revealed the presence of 17 phytocompounds of diverse classes like carbohydrates, fatty acyls, flavonoids, tannins etc. (**Table 10**; **Figure 6**). The first compound showed the characteristic protonated [M+Na]^+^ molecule at m/z 121.06, corresponding to hexenal (C_6_H_10_O), other major compounds detected were rosmarinic acid (polyphenol), digoxigenin monodigitoxoside (a steroidal saponin), methyl 4,6-di-O-galloyl-beta-D-glucopyranoside (a tannin) and others. One coumarin derivative named sagecoumarin (C_27_H_20_O_12_) was also detected at 11.86 min with a mass of 519.09 m/z. On the contrary, the methanolic extract of leaf-callus indicated the presence of only 6 phytoconstituents (**Table 11**; **Figure 7**). Among the compounds identified, 3 compounds belong to flavonoid group, 2 from steroidal group and one from terpenoid class. The compounds successfully identified were: Epicatechin 3-glucoside (453.13 m/z, [M+H]^+^); (15a,20R)-Dihydroxypregn-4-en-3-one 20-[glucosyl-(1->4)-6-acetyl-glucoside] (681.34 m/z, [M+H-H_2_O]^+^); Quercetin 3-glucoside 7-xyloside (597.14 m/z, [M+H]^+^); stigmasterol 3-O-beta-D-glucoside (597.41 m/z, [M+Na]^+^); Erythrodiol 3-decanoate (597.52 m/z, [M+H]^+^) and 3’,4’,5’-Trimethoxyflavone (313.10 m/z, [M+H]^+^).

#### 3.4.2 Analyses in the negative-ion mode

**Figure 11:**
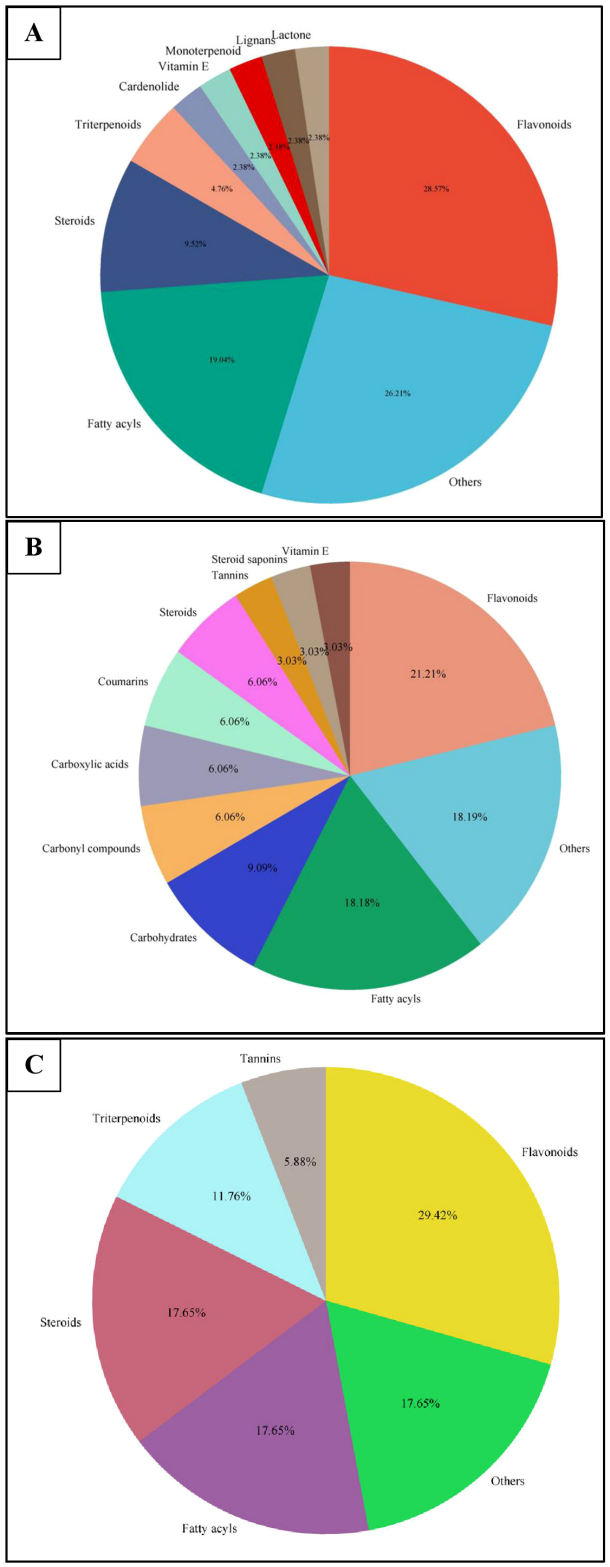
Pie-charts showing the proportions of the most abundant categories of phytocompounds detected in UPLC-ESI-QTOF-MS analysis of **(A)** *in vivo* grown leaf tissue, **(B)** *in vitro* grown leaf tissue, and **(C)** leaf derived callus of *D. purpurea* L.

Similar to positive ion, all the three samples were analyzed in negative ionization mode. Firstly, the field grown leaf sample was investigated and it showed some 22 bioactive compounds, more than observed in positive ion mode. The first compound eluted was 3,3’,4’,7-Tetrahydroxyflavan (flavonoid) at 0.70 min with the mass 273.07 m/z (**Table 12**; **Figure 8**). Likewise, multiple flavonoids such as epicatechin 3-glucoside (3.49 min), nepitrin (4.87 min), apigenin (7.35 min) were detected with the masses of 451.12 m/z, 477.10 m/z and 269.04 m/z, respectively. A base peak at m/z 425 was noticed, corresponding to the alpha-amyrin (a triterpenoid) at 5.21 min. Further analyses revealed that methanolic leaf (*in vivo*) extract contained several phytocompounds involving fatty acyls, steroids, triacyl glycerols, lignans and benzenoids. In a similar manner, the *in vitro* regenerated leaf extract was known to possess 16 phytoconstituents belonging to varied metabolite classes. 2-Hydroxypropyl glucosinolate (376.03 m/z), nepitrin (477.10 m/z), rhamnetin 3-laminaribioside (639.15 m/z), pregn-5-ene-3,20-dione-17-ol (329.21 m/z) and 2,3-Diphosphoglyceric acid (264.95 m/z) were some of the major compounds (**Table 13**; **Figure 9**). Finally, the callus extract of *D. purpurea* was also analyzed in negative ion mode and 11 bioactives were noted (**Table 14**; **Figure 10**). The characteristic compounds identified were squamoxinone C (fatty acyl) at 8.00 min, Delta4,16-Pregnadiene-3,20-dione at (steroid) at 10.29 min, 2,3-Diphosphoglyceric acid (carbohydrate) at 12.96 min with the masses corresponding to 595.45 m/z, 311.20 m/z and 264.95 m/z, respectively. Overall, the LC-MS analysis (both positive and negative ion mode) of the field grown leaf sample of *D. purpurea* had shown the presence of flavonoids in the major proportion (28.57%), followed by fatty acyl groups (19.04%), steroids (9.52%), triterpenoids (4.76%) and other compounds (26.21%) viz. the monosaccharide, saturated hydrocarbon, benzenoid etc. (**Figure 11A**). The *in vitro* raised leaf extract had the flavonoids as the highest occurring bioactive group (21.21%), followed by fatty acyls (18.18%), carbohydrates (9.09%), carbonyl compounds, carboxylic acids, coumarins and steroids (all at 6.06%), tannins (3.03%) etc. (**Figure 11B**). The major metabolite groups noted in callus extract followed the same trend as was noticed *in vivo*- and *in vitro* grown leaf tissue, with flavonoid being the top group (29.42%), then fatty acyl (17.65%), steroids (17.65%), triterpenoids (11.76%) and tannins (5.88%) (**Figure 11C**).

## 4 Discussion

To the best of our knowledge, this is the first study in characterizing metabolomes of cultured tissues in *Digitalis purpurea* by employing untargeted GC-MS and LC-MS approaches. Thus, in the current study, the leaf explants of *D. purpurea* were successfully used for callus induction and subsequent shoot regeneration (organogenesis) was achieved by amending MS medium with two most frequently used PGRs i.e. BAP and 2,4-D at varying concentrations. It was observed that a high BAP (8.8 µM) and low 2,4-D (0.9 µM) concentration exerted the best stimulatory effect on callus biomass production. Similar responses have earlier been reported in *D. purpurea* and several other related species (Verma et al., 2016b; Rad et al., 2021). On further sub-culturing, the callus became organogenic and produced green shoots on the same medium. Similarly, a relative higher concentration of BAP (compared to 2,4-D) was found to trigger organogenesis in various plant species such as *Pancratium maritimum* (Yasemin et al., 2023), *P. angulata and P. chenopodifolia* (Romo-Paz et al., 2023). On the contrary, several reports are available where promotive effects of BAP and NAA were noted for shoot organogenesis (de Oliveira et al., 2022; Raju et al., 2022; Liu et al., 2023).

Various internal and external factors like genetic makeup, used agronomic techniques, environmental conditions and *in vitro* culture practices often alter biochemical attributes in the wilds as well as *in vitro* raised plants (Phuyal et al., 2020).Thus, The biochemical and antioxidant markers levels were checked between mother (donor) plant and different *in vitro* raised tissues in *D. purpurea*. From biochemical assays, it was clearly evident that the *in vitro* grown tissues (both callus and organogenic derived shoot leaf) accumulated more phenolics and flavonoids than the donor *D. purpurea* plant. Higher content of phenols and flavonoids in the micropropagated tissues (compared to field grown plant) have also been reported in *Scrophularia takesimensis* (Jeong and Sivanesan, 2015) and *Dendrobium nobile* (Bhattacharyya et al., 2016). As PGRs utilized in *in vitro* culture system, regulate biosynthesis pathway genes, the plant tissue culture system plays a significant role in the production of phenols and flavonoids (Ghosh et al., 2018). The role of clonal propagation is receiving a lot of attention since the primary significance of medicinal plants as a natural source of antioxidants is realized (Bose et al., 2015). Plant tissue, rich in phenols and flavonoids is considered to be a good source of antioxidants as there is a positive correlation of phenolic, flavonoids with antioxidant activities (Khorasani Esmaeili et al., 2015). The antioxidant potential of mother tissues/plants and laboratory grown plants was evaluated by conducting DPPH, POD and SOD assays. DPPH is an easy, reliable and popular method for assessing the ability of plant extracts to scavenge radicals by quenching the stable purple colored DPPH radical into yellow colored DPPH (Aryal et al., 2019). SOD scavenges superoxide radical and manages the levels of H_2_O_2,_ while POD helps in oxidation and decomposition of H_2_O_2_ (Bansal et al., 2024). In our study, it was observed that the laboratory grown tissues (callus and *in vitro* leaf) possessed higher antioxidative activity than the field grown plant parts in all the three assays performed. These findings indicate a much greater antioxidant activity in micropropagated plant tissues compared to the mother plant, consistent with previous experimental observations from other researchers (Zayova et al., 2016; Ali et al., 2018; Mamgain et al., 2023).

Large-scale metabolite identification and/or quantification from one or more samples is referred to as untargeted metabolomic study. The metabolite profile strategy, otherwise called as top-down approach, examines the whole metabolomic profile of a given complicated sample rather than the requirement of a previous comprehensive hypothesis on a particular set of metabolites (de Souza et al., 2022). These can be achieved by performing untargeted GC-MS and LC-MS based metabolite profiling. The GC-MS technique has been applied in several plant species like *Tanacetum sinaicum* (Adel et al., 2021) and *Catharanthus roseus* (Bansal et al., 2023). Till date, there has been no previous untargeted metabolomics investigations in *D. purpurea* that examined *in vitro* regenerated callus and leaf tissue using GC-MS and LC-MS combined approach. In the present study, a comparative metabolite profile of *in vivo-, in vitro* grown leaf and leaf-callus has been made by using GC-MS and LC-MS techniques. The methanolic extracts of studied samples revealed the presence of more than 75 phytoconstituents belonging to various classes, like alkaloids, phytosterols, terpenoids, steroids, phenols, sugars etc. These detected bioactive compounds confer this plant diverse therapeutic importance. The major metabolites found in methanolic leaf extract of *in vivo* plant were 4-vinylguaiacol, lauric acid, myristic acid, loliolide, palmitic acid, squalene, stigmasterin, d-alpha-tocopherol etc. Loliolide is a type of monoterpenoid hydroxylactones, exhibiting anti-proliferative, anti-bacterial, anti-fungal and allelochemic activities and is reported in various plant species such as *Rauvolfia yunnanensis*,

*Veronica persica*, *Salvia divinorum* etc (Grabarczyk et al., 2015). Stigmasterin (or Stigmasterol) shows diverse range of pharmacological effects like anti-diabetic, immunomodulatory, antiparasitic, anticancer, anti-osteoarthritis, anti-inflammatory, antifungal properties (Bakrim et al., 2022). Squalene, a triterpenoid, possess antioxidant, anti-inflammatory, anti-neoplastic and anti-atherosclerotic properties (Lou-Bonafonte et al., 2018).

Similarly, the methanolic leaf extract of *in vitro* raised plant had 77 phytocompounds such as neophytadiene, phytol, beta-methylionone, linolenic acid, stearic acid, campesterol, gamma sitosterol, cycloartenol etc. Neophytadiene and phytol are diterpenoid compounds with anti-microbial, anti-inflammatory, anti-cancerous and antipyretic activities, which have been detected by GC-MS in various plant species (Willie et al., 2020; Banni and Jayaraj, 2022). Beta-sitosterol is widely known for its anti-diabetic, anti-inflammatory, cytotoxic and immunosuppressive properties (Salazar et al., 2020), campesterol (another sterol) has been associated with cancer prevention, anti-fungal and cholesterol lowering activities (Uttu et al., 2023). Callus extract on the other hand, showed a more varied range of metabolites such as linalool acetate, eugenol, cinnamic acid, jasmone, alpha-monostearin, 3-hydroxy-5-methoxyflavone, beta-amyrin, cycloartenol etc. in varied quantities. Eugenol (volatile phenolic compound) has been detected in several plant species, namely *Eugenia caryophyllata*, *Myristica fragrans*, *Ocimum basilicum* and act as antifungal, analgesic, anticancer, antiparasitic, antioxidant and antimicrobial agent (Abdou et al., 2021). Cinnamic acid is a key compound of aromatic carboxylic acid group, possessing anti-diabetic, anti-inflammatory, anti-cancerous and anti-microbial activity and is widely found in a number of plant varieties like *Cinnamomum cassia*, *Panax ginseng* etc (Ruwizhi and Aderibigbe, 2020). Beta-amyrin (a triterpenoid) demonstrates analgesic, anti-inflammatory, gastroprotective, hepatoprotective, anticonvulsant, antidepressive, antipancreatitic effects (Nogueira et al., 2018). This differential presence of metabolites in *in vitro* regenerated tissues (as compared to the mother plant parts) may be attributed to specific ecotype, genotype, explant, relative humidity, photoperiod, temperature, PGRs exposure and other variables in cultured conditions (Khan A. et al., 2021).

Finally, the methanolic extracts of each sample were subject to LC-MS analyses and the observation revealed that the *Digitalis purpurea* is enriched with a wide variety of phytocompounds such as flavonoids, fatty acyls, terpenoids, saponins, cardenolides, sugars, steroids, tannins and lignans, thereby increases plant’s medicinal potential. Several detected compounds in this present work, were reported previously in other Plantaginaceae members. These are like apigenin (269.04 m/z), Dicaffeoylquinic acid (517.13 m/z), luteolin (285.04 m/z), Quercetin 3-(2-caffeoylglucuronoside) (639.09 m/z) etc (Nedime et al., 2023; Bouali et al., 2024). The most significant phytocompound groups identified in both positive and negative ionization modes perhaps were flavonoids, fatty acyl and steroids. Major flavonoids detected in the current study include 3,3’,4’,7-tetrahydroxyflavan, nepitrin, luteolin, apigenin and kaempferid. Nepitrin has earlier been isolated from *Rosmarinus officinalis* and *Salvia plebeia* (Slimestad et al., 2022), both belonging to the same order Lamiales just like *Digitalis purpurea*. Kaempferid (299 m/z), luteolin (285 m/z) and apigenin (269 m/z) were also identified in the leaf extracts of *Digitalis trojana*. Flavonoids represent the most extensive category of naturally occurring compounds, with over 9000 phenolic chemicals identified in plants (Shomali et al., 2022). The application of flavonoids is quite widespread which includes antitumor, neuroprotective, anticancer, antibacterial, antiviral, antiangiogenic, antioxidant, and anti-proliferative activities (Ullah et al., 2020).

Fatty acyl followed by steroids group was the next most abundant metabolites detected in the present work. The fatty acyl group includes 2-hydroxylinolenic acid, ethyl stearate, octyl octanoate, dihydrojasmonic acid. Dihydrojasmonic acid is well known for its anti-cancerous, anti-depressant, anti-inflammatory, anti-nociceptive, anti-parasitic activities (Ghasemi Pirbalouti et al., 2014). 24-methylcycloartenol, delta4, 16-pregnadiene-3,20-dione, pregn-5-ene-3,20-dione-17-ol, campesterol 6’-hexadecanoylglucoside, stigmasterol 3-o-beta-d-glucoside were some of the major steroidal compounds identified in the tested samples. Campesterol and stigmasterol are the important phytosterols, found to be accumulated in *D. purpurea* tissues and act as precursor sterol for cardenolide biosynthesis (Carroll et al., 2023; Kunert et al., 2023). Two interesting derivatives of digoxigenin (and digoxin metabolite) i.e., digoxigenin monodigitoxoside and 20,22-dihydrodigoxigenin were detected in positive ion modes in *in vivo-* and *in vitro* grown leaf samples of *D. purpurea*. Their presence indicated that the cardenolides biosynthetic pathway was operational in studied experimental leaf tissue of both field grown and laboratory grown *D. purpurea.* Some other important metabolites identified were alpha-amyrin, rosmarinic acid, 2,3-diphosphoglyceric acid. Rosamarinic acid is a polyphenol, exhibiting antioxidant, anti-bacterial, anti-viral and anti-inflammatory activities (Andrade et al., 2018). In general, the callus extract showed the least number of phytocompounds in both positive and negative ionization modes as compared to the *in vivo-* and *in vitro*-grown leaves. This may be due to the fact that the callus is a simple tissue, some degree of cellular differentiation and tissue organization are necessary in controlling synthesis and accumulation of secondary metabolites (Karakas and Turker, 2016). These studies may pave the way for broad-spectrum drug development after ascertaining the bioactivity, toxicity and clinical trials of identified bioactive.

## 5 Conclusions

A comparative analysis of biochemical parameters, antioxidant activities and metabolite profiles of mother/donor plant and *in vitro* regenerated leaf tissues and leaf-callus were studied. To our knowledge, the current work represents the first metabolic profiling study of *in vitro* cultured tissues in *Digitalis purpurea* by GC-MS and UPLC-ESI-QTOF-MS techniques. The biochemical and antioxidant attributes showed that the *in vitro* derived tissues (callus and leaf samples) had higher level of phenols, flavonoids as well as antioxidant activities than the field grown (mother) plant. A variety of phytocompounds were identified and quantified in each sample by using GC-MS approach, revealing diverse pharmacological effects of this plant. The phytochemical composition of methanolic extracts of tissues were assayed by using UPLC-ESI-QTOF-MS in positive and negative modes. Major phytoconstituents detected were flavonoids, fatty acyls, steroids, triterpenoids, carbohydrates etc. The variation in metabolites of studied sample may be attributed to a number of factors like explant/tissue specific, genotype, used PGRs concentration, *in vitro* culture conditions etc. These analyses confirmed diverse therapeutic value of *D. purpurea;* the *in vitro* culture may therefore, be exploited for production of important bioactive compounds for pharmaceutical industry. Furthermore, studies like molecular docking and bio-prospecting, could be performed to deduce the ligand-protein interaction and biological properties of these therapeutically important phytocompounds, which will lead to pre-clinical and clinical trials in the later stages.

## Data Availability

The original contributions presented in the study are included in the article/supplementary material. Further inquiries can be directed to the corresponding author.
